# Unraveling the Complex Interplay between Epigenetics and Immunity in Alcohol-Associated Liver Disease: A Comprehensive *Review*

**DOI:** 10.7150/ijbs.87975

**Published:** 2023-09-04

**Authors:** Yali Liu, Tao Liu, Feiyu Zhang, Yanhang Gao

**Affiliations:** Department of Hepatology, The First Hospital of Jilin University, Jilin University, Changchun, Jilin, 130021, China.

**Keywords:** alcohol-associated liver disease, immune microenvironment, epigenetic modifications, exosomes, cytokines and chemokines

## Abstract

The mechanisms of immune dysfunction in alcohol-associated liver disease (ALD) have garnered growing research interest in recent times. Alcohol-mediated immune dysfunction has been implicated as a potential cause of ALD-associated microbial infection and inflammatory response. The immune microenvironment of an organism is essentially a complex network of interactions between immune cells, cytokines, extracellular matrix, and other immune-related molecules. This microenvironment is highly adaptive and responsive to environmental cues. Epigenetic reprogramming of the immune microenvironment has recently emerged as a key driver of ALD progression, particularly in the context of endotoxin tolerance and immune disorders. Although epigenetic modifications are known to play an important role in the regulation of the immune microenvironment in ALD, the specific mechanisms and molecular processes by which this regulation is achieved are yet to be fully understood. This paper aims to provide an overview of the current knowledge on the effects of alcohol consumption on epigenetics, with special focus on summarizing the data on the epigenetic regulatory mechanisms involved in the effects of alcohol consumption on the immune microenvironment. In addition, this paper aims to present a review of the epigenetic modifications involved in different forms of ALD. This review is expected to offer new perspectives for the diagnosis, treatment, monitoring, and prognostic assessment of ALD from an epigenetic perspective.

## Introduction

Alcohol is a well-known carcinogen, as classified by the World Health Organization. Chronic and excessive alcohol consumption can lead to tissue damage and numerous diseases, including alcohol-associated liver disease (ALD). The spectrum of ALD includes alcoholic fatty liver disease (AFLD), alcoholic hepatitis (AH), alcoholic liver fibrosis and cirrhosis, and alcoholic liver cancer 1. The morbidity and mortality rates of ALD have shown a steady year-on-year increase. Globally, the per capita alcohol consumption increased from 5.5 liters in 2005 to 6.4 liters in 2016 and is estimated to further increase to 7.6 liters by 2030 2. In parallel, the number and rate of alcohol-related deaths have increased by approximately 25% between 2019 and 2020, mainly due to the impact of the novel coronavirus epidemic in the last two years 3. In recent times, the mechanisms of immune dysfunction in ALD have become a major research hotspot 4, 5. The immune system contributes to the development and progression of ALD via two critical mechanisms: clearing foreign, gut-derived microbial byproducts through pathogen-associated molecular patterns and responding to tissue damage and cell death through damage-associated molecular patterns. The immune microenvironment is a complex network of molecules, cells, tissues, and organs that work together to protect the body from infectious agents and malignant cells. Epigenetic recoding of the immune microenvironment has recently garnered increasing research interest. While alcohol consumption, even in moderation, is known to affect the immune system, it is also known to extensively modulate liver epigenetics. Therefore, we believe that epigenetic alterations in the immune microenvironment caused by alcohol may be an important factor in the pathogenesis and progression of ALD.

Genetics and epigenetics together create the wonder of life that surrounds us. The term epigenetics refers to a collection of heritable and environmental changes that regulate gene expression without altering the DNA sequence. Epigenetic modifications include DNA methylation, histone modifications, microRNA-mediated gene silencing, and RNA methylation. These changes regulate the structure of secondary and tertiary DNA (chromatin), which in turn leads to an increase or decrease in the exposure of genes to transcription factors that up- or downregulate gene transcription, respectively. Factors that function as writers, readers, and erasers control chromatin structure through epigenetic modifications. Epigenetic modifications control the expression of many genes in the human body, and alcohol consumption can significantly alter epigenetic modifications, especially with respect to genes involved in immune regulation, promoting the development of ALD.

In this paper, we aim to provide a summary of the data on the epigenetic regulation of alcohol consumption, including alcohol metabolites, alcohol-metabolizing enzymes, and the methionine cycle. Furthermore, we review the current knowledge on the epigenetic regulation of the immune microenvironment, including immune cells, immune-related cells, immune mediators, and inflammatory pathways. Finally, this paper seeks to provide an innovative overview of the various forms of epigenetic modifications in ALD, providing new perspectives for the diagnosis, prognosis, and treatment of this disease.

## 1. The relationship between alcohol consumption and epigenetics

### 1.1. Alcohol metabolites (Fig. 1)

Once alcohol is consumed, it is primarily converted to acetaldehyde through the action of alcohol dehydrogenase (ADH), cytochrome P450 2E1 (CYP2E1), and catalase. The acetaldehyde thus produced is further converted to acetic acid by acetaldehyde dehydrogenase (ALDH). This acetic acid is synthesized by acetyl-CoA synthetase (ACS) into acetyl-CoA, which serves as a substrate for histone acetylation. Additionally, CYP2E1 can generate reactive oxygen species (ROS), which is also associated with histone H3 acetylation at Lys9. Acetaldehyde inhibits DNA methyltransferase activity, while ethanol increases the acetylation of H3-Lys9 by regulating histone acetyltransferase (HAT) and that histone acetylation may be the basis for ethanol-induced expression of the alcohol dehydrogenase 1 (*ADH1*) gene.

#### 1.1.1. Ethanol

Park et al. were the first to discover that intrahepatic ethanol metabolism induces acetylation of histone H3 at lysine 9 (H3-Lys9) 6, but not at Lys14. This may be partly because of the mechanism of ethanol metabolism. Subsequently, the same research group found that ethanol increases the acetylation of H3-Lys9 by regulating HAT and that histone acetylation may be the basis for ethanol-induced (*ADH1*) gene expression 7. The group further investigated this mechanism and found that long-term ethanol consumption selectively induces histone H3-Lys9 acetylation, specifically at the promoter and coding regions of the *ADH1* gene, despite increases in mRNA expression of the iNOS, c-jun, and *ADH1* genes. This site-specific acetylation was attenuated by the inhibition of ethanol metabolism by cyanamide and 4-methylpyrazole; however, overall histone acetylation was not enhanced by chronic ethanol treatment. These findings suggest that ethanol may induce the expression of different genes through distinct mechanisms and that further exploration may reveal the site-specific histone modifications that are triggered by ethanol 8. In short, epigenetic modifications of histone H3 through acetylation may be the underlying mechanism by which alcohol consumption induces *ADH1* expression *in vivo*.

#### 1.1.2. Acetaldehyde

ADH converts alcohol to acetaldehyde, which is known to be a highly toxic metabolite and a class I carcinogen 9. The *ALDH* gene is responsible for metabolizing acetaldehyde, and studies have found that *ALDH2* gene defects are prevalent in up to 40% of Asians. *In vitro* studies have previously shown that acetaldehyde can form numerous adducts with proteins and nucleic acids, causing cellular damage and triggering immune responses. Acetaldehyde is also known to cause various epigenetic modifications. For example, it has been shown to inhibit DNA methyltransferase activity *in vitro* 10. A recent study on acetaldehyde and hepatitis C virus (HCV) revealed that acetaldehyde enhances HCV-induced inhibition of STAT-1 methylation, which ultimately leads to the blocking of the interferon signaling that mediates innate immunity. In terms of mechanism of action, acetaldehyde is likely to have a direct inhibitory effect on DNA and histone methyltransferase 11. Additionally, acetaldehyde affects histone acetylation, and its accumulation in excess amounts reduces ethanol-induced histone acetylation, possibly because it needs to be further metabolized (e.g., to acetic acid) 6. Therefore, further research on the intrinsic link between acetaldehyde and epigenetic modifications is imperative to identify corresponding targets that can reduce the damage caused by acetaldehyde to the body.

#### 1.1.3. Acetic acid

Acetaldehyde dehydrogenase converts acetaldehyde into acetic acid—a less reactive product that can be further converted to acetyl coenzyme A by ACS. Acetyl coenzyme A then serves as a substrate for histone acetyltransferases, which in turn catalyze the acetylation of histone lysine residues in the liver 12. Thus, alcohol metabolism can directly induce histone acetylation. To investigate this phenomenon, Kriss and colleagues 13 studied the effect of ethanol metabolism on histone acetylation in mouse liver using ^13^C_2_-labeled ethanol and mass spectrometry. Their findings showed significant incorporation of ethanol-derived ^13^C_2_ into the N-terminal lysine acetylation site at histones H3 and H4. Notably, a significant increase in site-specific histone acetylation occurred four hours after ethanol ingestion, following which there was a return to baseline levels at 24 hours. This suggests that the site-specific admixture of ethanol in histone acetylation via its metabolite acetic acid may be the result of a specific and transient transcriptional regulatory response to acute ethanol exposure. Future studies should further explore how acetic acid redistribution and utilization *in vivo* affect other tissues.

### 1.2. Alcohol-metabolizing enzymes

#### 1.2.1. ADH

Upon ingestion, ethanol is quickly absorbed by the stomach and intestines and subsequently transported to the liver for metabolism. The initial biochemical reaction involves the conversion of ethanol to acetaldehyde, which is primarily mediated by alcohol dehydrogenase and, to a lesser extent, by CYP2E1 and catalase 14. Different tissues exhibit different expression forms of alcohol dehydrogenases, including ADH1A, ADH1B, and ADH1C. Dannenberg and his team 15 have shown that the methylation of *ADH1A*, *ADH1B*, and *ADH1C* occurs in HepG2 cells and that gene repression resulted from methylation at the upstream regions of these genes. Furthermore, they showed that the inhibition of DNA methylation using decitabine resulted in the upregulation of *ADH1B* and *ADH1C*, whereas the inhibition of histone deacetylases (HDACs) using trigonelline A promoted *ADH1C* expression. Put together, these findings indicate that hepatocyte epigenetic methylation and deacetylation mechanisms regulate the alcohol metabolism genes *ADH1B* and *ADH1C*. However, the alcohol-induced changes in the liver-specific epigenetic regulation of these genes still warrant further in-depth investigation.

#### 1.2.2. ALDH2

The regulation of ALDH2 plays a crucial role in the development and progression of alcoholic hepatocellular carcinoma (HCC) via epigenetic mechanisms. Studies on *ALDH2*-deficient mice have shown that hepatocytes can produce harmful oxidized mtDNA in large quantities, which are then transferred to adjacent hepatocytes via extracellular vesicles. This transfer subsequently co-activates multiple oncogenic pathways (such as JNK, STAT3, BCL-2, and TAZ) via acetaldehyde, thereby promoting alcohol-associated HCC 16. ALDH2 exhibits tumor-suppressive effects in HCC. Overexpression of ALDH2 effectively inhibits the proliferation, migration, and invasion of HCC cells. miR-671-5p, which acts as an upstream regulatory gene of ALDH2, is significantly overexpressed in HCC and has a negative regulatory relationship with ALDH2. Forced expression of miR-671-5p leads to the promotion of the proliferation, migration, and invasion of HCC by inhibiting ALDH2 17. In addition, DNA methylation and histone modifications also regulate the expression of ALDH2, thereby promoting the development and progression of ALD. The latest research findings indicate a significant discovery 18: hypomethylation at the *ALDH2* gene occurs in individuals with cirrhosis who engage in excessive alcohol consumption, unlike the case in those without cirrhosis. Furthermore, DNA hypermethylation at the promoter regions of ALDH2 has been shown to be closely linked to an elevated risk of alcohol use disorder (AUD) in males of Chinese Han ethnicity in Yunnan Province 19. This valuable insight sheds light on the mechanisms underlying epigenetic regulation in ALD. A recent study has also shown that ALDH2 mediates the anti-senescent effect of ethanol by promoting SIRT1 nuclear translocation and thereby enhancing p53 deacetylation 20. These findings highlight the crucial role of epigenetic modifications involving ALDH2 in the development of ALD. It is imperative to delve further into the intricate interplay between the presence of ALDH2 deficiency or ALDH2 variants and the realm of epigenetics within the context of ALD.

#### 1.2.3. CYP2E1

Chronic alcohol consumption not only oxidizes ADH and ALDH, but also stimulates the hepatic endoplasmic reticulum, causing the synthesis of CYP2E1, which in turn initiates the hepatic microsomal ethanol oxidation system. CYP2E1 metabolizes ethanol and generates ROS, which trigger liver inflammation and hepatocyte necrosis. ROS are known to be hepatotoxic due to their ability to react with macromolecules, inactivate enzymes, cause DNA damage, modify proteins, induce lipid peroxidation, and trigger histone acetylation. Choudhury and his colleagues 21, in their experiment using rat hepatocytes treated with ethanol, found that the production of ROS is linked to histone H3 acetylation at Lys9. The administration of ROS scavengers, such as N-acetylcysteine, significantly mitigated the effect of alcohol-induced ROS and histone H3 acetylation, whereas the administration of ROS inducers and inhibitors of mitochondrial complexes I and III (rotenone and antimycin, respectively) enhanced histone H3 acetylation at Lys9. These findings provide the first evidence of the crucial role of ROS in alcohol-induced histone acetylation, thus identifying a new therapeutic target to prevent oxidative stress in ALD.

### 1.3. The relationship between alcohol consumption and methionine metabolism

**Figure 2:**
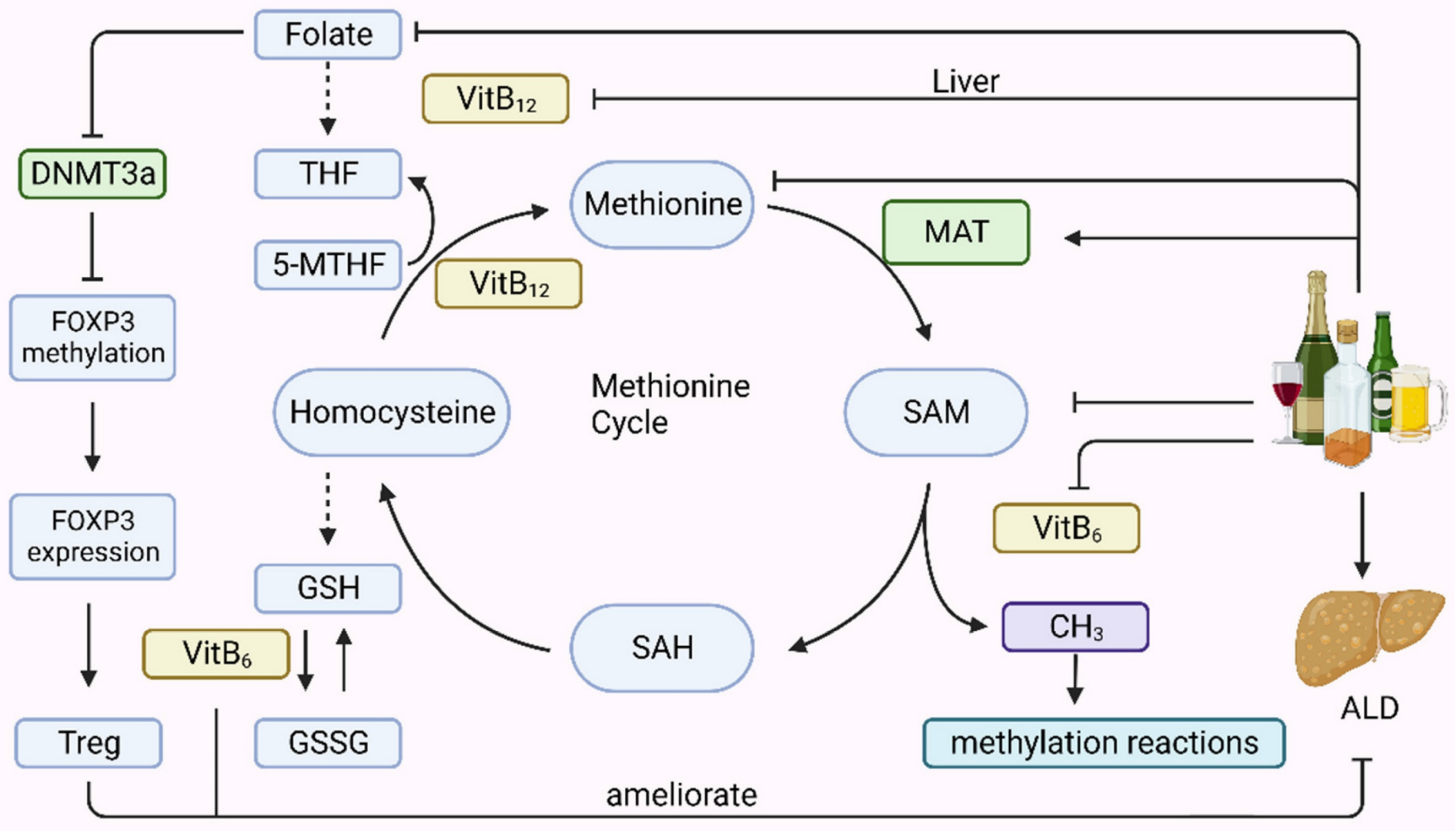
** The association of alcohol consumption with the methionine cycle.** Methionine metabolism primarily occurs in the liver and involves two main components: the transmethylation cycle, which generates S-adenosylmethionine (SAM) and its subsequent metabolism into homocysteine (SAH), and the transsulfuration pathway, which produces reduced homocysteine into glutathione (GSH). These processes require the involvement of folic acid, vitamin B6, and vitamin B12. Drinking alcohol is intricately linked to the methionine cycle, which generates methyl groups that serve as substrates for DNA and histone methylation in epigenetics. Alcohol consumption can alter epigenetic modifications by affecting the intermediates, enzymes, and cofactors involved in the methionine cycle. It reduces the levels of folate, methionine, SAM, vitamin B6, and vitamin B12 in the body. Ethanol consumption upregulated MAT1A and MAT2A gene expression. Folic acid can lead to a decrease in DNMT3a expression, which downregulates the methylation level of the forkhead Box P3(FOXP3) promoter region, thereby increasing the abundance of FOXP3 expression, an important transcription factor for Treg cells, which can alleviate liver inflammatory injury in ALD. 5-MTHF, 5-methyltetrahydrofolate; DNMT, DNA methyltransferase; FOXP3, forkhead Box P3; GSH, glutathione; GSSG, oxidized glutathione; MAT, methionine adenosyltransferase.; SAH, S-adenosylhomocysteine; SAM, S-adenosylmethionine; THF, tetrahydrofolate; Treg Regulatory T cell

Methionine metabolism primarily occurs in the liver and involves two main components: the transmethylation cycle, which generates S-adenosylmethionine (SAM) and its metabolite S-adenosylhomocysteine (SAH), and the trans-sulfuration pathway, which produces reduced homocysteine into glutathione (GSH) (Fig. 2). Both these processes require the involvement of folic acid, vitamin B6, and vitamin B12. Drinking alcohol is intricately linked to the methionine cycle, which generates methyl groups that serve as substrates for DNA and histone methylation occurring during epigenetic modification. Alcohol consumption can alter epigenetic modifications by affecting the intermediates, enzymes, and cofactors involved in the methionine cycle. Alcohol reduces the levels of folate, methionine, SAM, vitamin B6, and vitamin B12 in the body. Ethanol consumption upregulates the expression of *MAT1A* and *MAT2A* genes. Folic acid can lead to a decrease in DNA methyltransferase 3a (DNMT3a) expression, which downregulates the methylation level of the forkhead box P3 (FOXP3) promoter region, thereby increasing the expression of FOXP3, an important transcription factor for Treg cells that can alleviate liver inflammatory injury in ALD.

#### 1.3.1. The relationship between abnormal methionine metabolism and ALD

The SAM to SAH ratio is a useful indicator of methylation capacity 22. Growing evidence suggests that ethanol exposure can alter DNA and histone methylation. Methionine adenosyltransferase(MAT) is a liver-specific enzyme involved in SAM synthesis. Studies in rats by Lu and colleagues revealed that ethanol upregulated the expression of the *MAT1A* and *MAT2A* genes, with only MAT2A protein levels increased. Furthermore, administration of ethanol led to a reduction in hepatic methionine and SAM levels, resulting in decreased DNA methylation and increased DNA strand breaks 23. Reduced hepatic SAM levels can lead to liver injury through impaired antioxidant defense mechanisms and abnormal epigenetic regulation of genes involved in alcoholic liver injury. Thus, ethanol-induced abnormalities in methionine metabolism play a crucial role in the pathogenesis of ALD.

#### 1.3.2. The relationship between folic acid deficiency and ALD

Folic acid is converted by dihydrofolate reductase to tetrahydrofolate, which can act as a carrier for one-carbon units, such as methyl groups, to form methyltetrahydrofolate. This methyl group can then be transferred to homocysteine, to form methionine. The liver is the primary storage site for folate in the body 24. A clinical study revealed that the amount of folate stored in the liver is significantly reduced in patients with ALD; this reduction may be attributed to the reduced vitamin intake or abnormal intrahepatic metabolism associated with alcohol consumption 25. Folic acid, as a major source of methyl donors for DNA methylation, has been shown to be beneficial in regulating inflammation, which is commonly impaired in ALD. A recent study 26 demonstrated that folic acid can limit ethanol-induced inflammatory damage by increasing the expression of hepatic Treg cells. This is achieved by a folic acid-induced decrease in DNMT3a expression, which downregulates the methylation level of the forkhead box P3 (FOXP3) promoter region, thereby increasing the FOXP3 expression—an important transcription factor for Treg cells. The increased expression of FOXP3 counters the Th17/Treg imbalance. Taken together, these findings suggest that folic acid supplementation may alleviate liver inflammatory injury by improving Th17/Treg imbalance and could represent a viable strategy for preventing ALD.

#### 1.3.3. The relationship between vitamin B6 and vitamin B12 deficiency and ALD

Vitamin B6 is a coenzyme that catalyzes the catabolism of homocysteine to cystathionine, which is a cysteine that eventually generates GSH and serves as an important reducing agent that protects against oxidation of sulfhydryl groups in protein or enzyme molecules. Approximately two-thirds of cases of chronic alcoholism with liver disease have vitamin B6 deficiency, and overall levels are significantly lower than those in normal subjects 27. The most widely accepted cause of vitamin B6 deficiency is its degradation by acetaldehyde products of alcohol metabolism and subsequent urinary waste of the free vitamin 28. Vitamin B12, on the other hand, is a coenzyme of transmethylesterase, which regulates the transmethylation reaction during SAM production. Although intestinal absorption of vitamin B12 is impaired in chronic alcoholism, circulating levels of vitamin B12 are frequently elevated in ALD 29. However, a recent study showed that hepatic vitamin B12 levels were reduced in chronic alcoholism, although elevated serum levels of vitamin B12 and its analogs remain elevated, thereby suggesting that damaged hepatocytes are unable to retain vitamin B12 reserves 30. When both vitamins B6 and B12 are deficient, homocysteine buildup can cause hyperhomocysteinemia, which in turn increases the risk of atherosclerosis, thrombosis, and hypertension. Recent studies have also shown that hyperhomocysteinemia compromises the autophagic capacity of Stx17. Supplementation with vitamin B12/folic acid may restore the autophagic capacity of Stx17 and reduce inflammation and fibrosis in patients with non-alcoholic steatohepatitis (NASH) and may represent a new option as first-line treatment for NASH 31. Since NASH and ALD share many features, vitamin B supplementation can be considered as a potential epigenetic modulatory approach for treating ALD.

## 2. Epigenetic regulatory mechanisms of alcohol consumption on the immune microenvironment (Fig. 3)

Alcohol consumption can impact the occurrence and progression of ALD by modulating immune cells and immune-related cells such as macrophages, neutrophils, hepatic stellate cells (HSCs), T lymphocytes, natural killer T (NKT) cells, and liver sinusoidal endothelial cells (LSECs) through epigenetic modifications (DNA methylation, histone modifications, microRNA). These immune factors, in turn, influence the levels of certain cytokines, chemokines, and extracellular vesicles involved in ALD.

### 2.1. Immune cells

#### 2.1.1. Macrophages

##### 2.1.1.1. DNA methylation

Alcoholic liver injury is characterized by a macrophage-mediated inflammatory response, with gene silencing regulated by DNA methylation playing a key role in the inflammatory process. Recent research has shown that mononuclear cells have the ability to infiltrate liver tissue and convert into macrophages derived from mononuclear cells, thereby playing an important role in repair following acute liver necrosis 32. These transformed macrophages can also respond to the activation of peptidoglycans and toll-like receptor 2, leading to the release of IL-1β and IL-8, thus promoting the occurrence and development of changes associated with ALD 33.

Ethanol consumption induces aberrant DNA methylation patterns and upregulates the protein expression of DNMT1, DNMT3a, and DNMT3b. DNMT3a-mediated methylation of the proline-serine-threonine phosphatase interacting protein 2 (PSTPIP2) has been recently shown to amplify the inflammatory response in alcoholic liver injury via the regulation of the STAT1 and NF-κB pathways. PSTPIP2, a protein that is mainly expressed in immune cells such as macrophages, plays a crucial role in ALD 34. Liver-specific recombinant AAV serotype 9 (rAAV9)-mediated overexpression of PSTPIP2 in ethanol-fed mice significantly alleviated liver injury and inflammation; on the other hand, silencing PSTPIP2 exacerbated the alcohol-induced inflammatory response *in vitro*. In terms of the underlying mechanism, PSTPIP2 downregulation in ALD may be linked to DNA methylation, with DNMT3a binding directly to the PSTPIP2 promoter and inhibiting PSTPIP2 expression in macrophages. To summarize, alcohol consumption can modify PSTPIP2 expression in macrophages through DNMT3a-mediated methylation, thereby contributing to the inflammatory response in ALD via the STAT1 and NF-kB signaling pathways.

Zinc-finger swim-type-containing 3 (ZSWIM3) is a novel SWIM zinc-finger chelating structural domain that is expressed in immune cells, particularly macrophages. ZSWIM3 expression is consistently reduced in macrophages isolated from the livers of ethanol-fed mice. Overexpression of ZSWIM3 has been shown to attenuate ethanol-induced liver injury and suppress inflammatory responses *in vivo*. Forced expression of ZSWIM3 has been shown to have anti-inflammatory effects *in vitro*. In terms of mechanism of action, alcohol exposure reduces ZSWIM3 expression in Kupffer cells, possibly due to DNMT3b-induced hypermethylation of the ZSWIM3 promoter. Downregulation of ZSWIM3 activates the bridging protein tumor necrosis factor receptor-associated factor 2 (TRAF2), which plays a crucial role in the activation of the NF-kB signaling pathway and subsequent increase in the transcription of IL-1b, TNFα, IL-6, and the monocyte chemoattractant protein-1 (MCP-1) as well as the protein production of IL-1b, TNFα, and IL-6 35. These changes trigger a pro-inflammatory phenotype. Therefore, it can be inferred that the induction of liver-specific expression of PSTPIP2 and ZSWIM3 may negatively regulate macrophage activation and inhibit the release of inflammatory factors, thereby blocking the inflammatory process in ALD.

##### 2.1.1.2. Histone and histone post-translational modifications

Prolonged exposure to alcohol can cause modifications to the histones in macrophages, which result in enhanced pro-inflammatory responses. A recent study on the epigenetic modifications of tissue-resident macrophages showed that chronic heavy alcohol consumption; led to an increase in the levels of the histone marker H3K4me3 36. However, there were no significant differences in the levels of mono- or dimethylation of the macrophage histone H3K4, while the levels of H3K9me3 remained unchanged. These findings suggest that chronic heavy alcohol consumption leads to alternations in the immune adaptability of tissue-resident macrophages through epigenetic mechanisms.

Enhancer of Zeste homolog 2 (EZH2) is a protein with histone methyltransferase activity; it mainly exerts transcriptional repression through specific methylation modification of lysine at the H3K27 site of histone. A study has shown that systemic EZH2-catalyzed H3K27me3 expression increases during liver failure, while its enrichment on the TNFα promoter in Kupffer cells decreases. This suggests that EZH2 enhances the production of pro-inflammatory cytokines released by macrophages after stimulation during liver failure 37. However, the patients with acute-on-chronic liver failure (ACLF) in that study were not ALD-ACLF patients. Therefore, further studies are needed to investigate the altered epigenetic modifications of macrophages specifically in ALD-ACLF patients.

HDAC11 is a member of the histone deacetylase family that has been shown to negatively regulate the expression of the gene encoding interleukin 10 (IL-10) in antigen-presenting cells 38. Recent research has shown that HDAC11 level is significantly elevated, while IL-10 level is significantly decreased in Kupffer cells isolated from alcohol-fed mice 39. Knockdown of HDAC11 in mice using small interfering RNA has been shown to increase IL-10 secretion by alcohol-pretreated macrophages, thereby leading to the attenuation of the hepatic inflammatory response. To establish a link between experimental observations and clinical outcomes in ALD patients, future research is warranted to determine whether chromatin-related changes can alter the effector functions of macrophages, including pathogen killing, phagocytosis, and wound healing. An understanding of these changes could help identify new epigenetic targets for the treatment of ALD patients.

##### 2.1.1.3. microRNA

Alcohol-induced miRNAs, particularly, miR-155 and miR-122, affect the function of monocytes/macrophages, including oxidative stress, phagocytosis, and angiogenesis. A series of studies have shown that miR-155 plays a crucial role in ALD 40. Elevated expression of miR-155 has been observed in ethanol-fed mice and alcohol-treated RAW264.7 macrophages. MiR-155 inhibits the negative regulators of the TLR4 pathway, thereby reducing their expression, promoting macrophage sensitivity to lipopolysaccharide (LPS), and increasing the production of pro-inflammatory TNFα in the liver, thus aggravating the inflammatory response 39. In addition, miR-155 downregulates STAT3 and SOCS1 in mouse macrophages, resulting in the upregulation of pro-inflammatory cytokines TNFα and IL-1b and the downregulation of anti-inflammatory cytokine IL-10 41. Studies have shown that as compared to control mice, miR-155 gene knockout (KO) mice have significantly less severe liver damage, steatosis, inflammation, and fibrosis from alcohol exposure 42. In addition to regulating the gene expression within the cells that produce them, miRNAs can also modulate the function of other cell populations, including monocytes and macrophages, through paracrine signaling via extracellular vesicles 43. MiR-122 is a liver-specific miRNA that is associated with lipid metabolism, stress response, and HCV replication 44. Notably, circulatory levels of miR-122 have been shown to be increased in both chronically alcohol-fed mice and alcohol-dependent patients. *In vitro*, miRNA-122 is transferred from ethanol-treated liver cells to monocytes via extracellular vesicles, resulting in the upregulation of pro-inflammatory cytokines TNFα and IL-1b 45. MiR-34a is the most upregulated miRNA in the liver of ethanol-fed mice and is a liver-specific miRNA known to be involved in liver pathology. Recent studies have shown that miR-34a deficiency inhibits the activation of liver macrophages and production of cytokines, as well as the infiltration of macrophages and neutrophils, thereby leading to a reduction in angiogenesis and liver inflammation. Therefore, inhibition of miR-34a may be a new therapeutic approach to protect the liver from inflammation and fibrosis 46. To sum up, miRNAs have a powerful regulatory effect on macrophage function, and modulating macrophages through microRNA regulation may be a potential therapeutic strategy for reducing the severity of inflammation in ALD.

##### 2.1.1.4. RNA methylation

The most prevalent internal RNA modification within eukaryotic cells is m6A; this modification plays a pivotal role in the emergence and progression of ALD. This modification, known as N6-methyladenosine (m6A), is derived from the m6A methyltransferase complex. A recent study investigated the involvement of the m6A-associated enzyme, methyltransferase-like 3 (METTL3), in ALD 47. In that study, Kupffer cells isolated from the livers of mouse models of alcoholic steatohepatitis (ASH) showed pyroptosis triggered by alcohol consumption, which led to an elevated release of IL-1β, an inflammatory cytokine. *In vitro* experiments with lentivirus-mediated silencing of METTL3 in BMDMs and RAW264.7 cells effectively demonstrated that METTL3 had the capacity to mitigate pyroptosis by influencing the splicing of pri-miR-34A, a precursor to a microRNA. To summarize, the inhibition of METTL3 contributed to a reduction in the inflammatory cytokine surge resulting from Kupffer cell pyroptosis in ASH mice. It is imperative to acknowledge that the knowledge on RNA methylation is continually advancing, and although a mounting body of evidence connects m6A modifications to various diseases, including ALD, the precise mechanisms and consequential effects of these effects are still under rigorous investigation.

#### 2.1.2. Neutrophil

Neutrophils serve as short-lived innate immune cells that constitute the first line of defense against microbial infections. To protect the host against invading pathogens, these cells utilize various defense strategies, such as releasing effector molecules via degranulation, phagocytosis, and the production of neutrophil extracellular traps (NETs) 48. In addition to mediating the release of immune-activating or suppressive cytokines, neutrophils play a vital role in regulating the innate and adaptive immune systems. A recent study has shown how neutrophilia can lead to liver injury in patients with alcoholic hepatitis: neutrophils in AH patients become activated and produce NETs, resulting in liver injury and an increase in the expression of functionally defective low-density neutrophils (LDNs). Transcriptome analysis has revealed that high-density neutrophils (HDNs) and LDNs have opposite transcriptome profiles. Unlike activated HDNs, LDNs exhibit a functionally failing/deficient phenotype 5. Researchers The exact mechanism by which alcohol induces NETs and produces functionally defective LDNs still remains elusive. It is also unclear whether neutrophils regulate their functional activity through intrinsic epigenetic mechanisms. Therefore, future studies should focus on exploring the interactions between NETs and epigenetic modifications to identify more effective molecular targets in patients with AH.

As an essential component of the granulocyte lineage, PU.1 is a critical transcription factor in the hematopoietic system, particularly in the bone marrow. A recent study has shown that PU.1's main function is to broadly inhibit enhancer accessibility by recruiting HDACs. This action suppresses the exaggerated immune response of neutrophils 49. Thus, the neutrophil-dependent inhibitor program mounted on PU.1 protects the host from an excessive intrinsic immune response through epigenomic modifications. Brahma-related gene 1 (Brg1) is a core catalytic subunit of the chromatin-remodeling complex that has been shown to be involved in the pathogenesis of ALD; it promotes neutrophil infiltration via the stimulation of the hepatocyte-derived chemokine CXCL14 50. Given that AH is characterized by neutrophil infiltration 51, exploration from the point of view of epigenetics are necessary to determine the mechanisms underlying liver injury caused by neutrophilia in AH patients.

#### 2.1.3. Lymphocyte

The role of hepatic lymphocytes in the pathogenesis of ALD has not received much attention thus far. In a recent study, the functional dysregulation between liver innate lymphoid cells (ILC1) and NK cells was found to cause a significant increase in IL-17 levels, thereby promoting the progression of alcohol-related fatty liver disease 52. Similarly, T-cell activation is closely associated with epigenetic regulation. The T-cell receptors (TCRs) on the cell surface play a crucial role in promoting T-cell proliferation and differentiation by upregulating DNA and histone methylation via the methionine cycle. Histone methylation complex expression is low in naive T cells and increases upon T-cell activation. This suggests that T cells require a steady supply of methionine to maintain their activated state. However, TCRs can only upregulate methionine transport and provide methyl donors in response to antigens. This enables dynamic nucleotide methylation and epigenetic reprogramming, which drive T-cell differentiation 53. Methionine has been shown to play a crucial role in epigenetic reprogramming in CD4^+^ T helper cells. Metabolomic analyses have shown that activated T cells use exogenous methionine to synthesize the universal methyl donor, SAM. Furthermore, methionine restriction has been found to reduce histone H3K4 methylation (H3K4me3) in the promoter region of key genes involved in Th17 cell proliferation and cytokine production, thereby reducing the proliferation and function of pathogenic Th17 cells 54. Thus, dietary methionine restriction may hold promise as a metabolic approach toward the management of autoimmune diseases by causing the inhibition of pathogenic Th17 cells. Alcohol intake has also been found to induce a pro-inflammatory shift in the immune cell population in the gut, including changes such as an increase in Th17 cells. The abovementioned study results suggest that restricting methionine in the diet could reduce Th17 cell activation, which could alleviate alcohol-induced liver injury and reverse the intestinal inflammation associated with alcohol consumption. Additionally, histone methyltransferase G9a inhibitors have been recently shown to regulate intestinal inflammation by enhancing cholesterol metabolism in CD4^+^ T cells, leading to the development of Tregs *in vivo* and *in vitro* 55. Overall, modulating T-cell activity through epigenetic mechanisms to alleviate the inflammatory response holds promise as a potential therapy for ALD.

### 2.2. Immune-related cells

#### 2.2.1. Hepatic stellate cell

Liver fibrosis is a complex process that involves the activation and transdifferentiation of HSCs into myofibroblasts. Metabolic reprogramming has emerged as a crucial factor in the activation of fibrotic cells across various organs. DNA and histone methylation is key to the activation of HSCs. In liver fibrosis, excessive deposition of extracellular matrix (ECM) components occurs, and calcium-regulated neurophosphatase (CaN) plays a crucial role in ECM accumulation. Regulator of calcium-regulated neurophosphatase 1 (RCAN1) is an endogenous inhibitor of CaN. A recent study 56 revealed that RCAN1.4 expression is selectively downregulated in liver fibrosis. Decitabine and DNMTs-RNAi inhibitors restore RCAN1.4 expression, indicating that DNA methylation causes the reduction in RCAN1.4 expression. ChIP analysis confirmed that DNMT1 and DNMT3b induced methylation of the RCAN1.4 promoter. Furthermore, overexpression of RCAN1.4 promotes the apoptosis of activated HSCs, both *in vitro* and *in vivo*, and also attenuates liver fibrosis. Thus, inhibiting DNA methylation to upregulate RCAN1.4 may serve as a potential therapeutic strategy for treating alcoholic liver fibrosis. Studies have demonstrated that methylation of histone H3 at lysine 4, lysine 9, and lysine 27 is a critical and dynamic epigenetic modification occurring during HSC activation. The trimethylation of histone H3 (H3K4me3) at lysine 4 is typically associated with gene activation, while H3K9me2/3 and H3K27me2/3 often lead to gene repression 57. Recent investigations have examined the differential expression and opposing functions of histone H3K27 methylesterase EZH2 and demethylase JMJD3 in the activation of HSCs and liver fibrosis. EZH2 and JMJD3 were found to play opposite roles in the regulation of HSC activation and that DZNep inhibition of EZH2 or siRNA silencing suppresses the activation and proliferation of HSCs, while JMJD3 overexpression also achieves these effects. Furthermore, demethylation in all of the above scenarios attenuated liver fibrosis 58. That study is among the few to investigate the role of methyltransferases and demethylases in the epigenetic modification of histones in liver fibrosis. G9a is an H3K9 methyltransferase, and G9a and DNMT1 are expressed together in fibrotic cells after HSC activation. This expression of G9a and DNMT1 is required for TGF-1-induced activation of HSC fibrosis, and the first dual G9a/DNMT1 inhibitor, CM272, represents a novel pharmacological agent for targeting DNMT1 and G9a to counteracts the TGF-1-induced pro-fibrotic response and metabolic reprogramming of HSCs 59. Taken together, these findings suggest that targeting epigenetic modifications, specifically in HSCs, could be a potential therapy for reversing liver fibrosis.

#### 2.2.2. Liver sinusoidal endothelial cell

LSECs contribute to maintaining hepatic homeostasis by performing multiple functions, including blood filtration to remove pathogens, supplying oxygen and nutrients to hepatocytes, metabolizing drugs and alcohol, and regulating immunity 60. LSEC dysfunction has been observed in various liver diseases, including ALD. LSECs express key enzymes, such as CYP2E1 and ADH1, that are involved in alcohol metabolism 61. Under normal circumstances, ADH1 can promote Hsp90 acetylation to some extent by metabolizing alcohol, without affecting LSEC function. However, ADH1 induction in LSECs does not occur in the presence of alcohol excess, and the upregulation of CYP2E1 increases Hsp90 acetylation, which reduces its interaction with eNOS, thus leading to decreased NO production—a typical manifestation of LSEC dysfunction. The liver can be protected from inflammation by eNOS-derived NO, which inhibits Kupffer cell activation. Excessive alcohol consumption can lead to acetylation of Hsp90 and its reduced interaction with eNOS, which are potential mechanisms of LSEC dysfunction and liver injury 62. Thus, reducing Hsp90 acetylation through overexpression of AAV gene-delivered HDAC isozyme (HDAC6) can increase Hsp90-eNOS interaction and restore eNOS-derived NO production, thereby ameliorating LSEC dysfunction and liver inflammation. Therefore, HDAC6-specific overexpression in AAV8-mediated gene delivery of LSECs may represent an innovative therapeutic strategy for ALD.

### 2.3. Immune mediators

#### 2.3.1. Cytokines

There is ample evidence of an abnormal circulating cytokine profile in ALD, which is attributed to the stimulation of peripheral pro- and anti-inflammatory cytokine secretion by immune cells upon chronic alcohol consumption. This abnormal cytokine profile may vary with the stage of the disease 63. Various cytokines play distinct roles in ALD. IL-20 and IL-22 are members of the IL-10 family. IL-10 serves as a well-established anti-inflammatory cytokine, and IL-22 provides epithelial protection and antimicrobial function, thus demonstrating therapeutic potential for protection against organ damage 64-67. However, recent research indicates that IL-20 may exacerbate liver inflammation and bacterial infections 68. Therefore, downregulation of IL-20 and upregulation of IL-22 through acquired epigenetic mechanisms may offer represent viable therapeutic approaches toward combating ALD.

AH is known to be associated with a robust cholestatic and biliary response 69. Furthermore, biliary disease is marked by substantial biliary fibrosis, a critical regulator of which is the downstream signaling of TGF-β. In the liver, KAT2A—a specific lysine acetyltransferase—is expressed primarily by bile duct cells, whereas KAT2B is predominantly expressed in parenchymal hepatocytes, such as hepatocytes. KAT2A-mediated H3K9AC is responsible for the epigenetic activation downstream of TGF-β and may selectively regulate the gene network of fibrosis activated by HSCs, without affecting other functions of TGF-β. Consequently, inhibitors specific to KAT2A could be used to selectively target bile duct cells to prevent bile duct fibrosis, without impacting other hepatocytes 70. Thus, epigenetic regulation of TGF-β expression may represent a viable therapeutic option for the prevention of biliary response and biliary fibrosis in ALD.

#### 2.3.2. Chemokines

Chemokines are a group of small signaling proteins that are secreted by cells. They can be classified into four families: CXCLs, CCLs, CX3CLs, and XCLs4. Their primary function is to chemotactically attract and activate immune cells. Chemokines have been shown to play important roles in various biological processes, both in health and disease. With the advancement of functional genomics, chemokine transcriptional regulation has attracted increasing research interest 71. Alcohol consumption can induce various physiological changes and bodily responses that may impact the production and function of chemokines. Elevated levels of CCL2, CXCL5, and CXCL6 are positively associated with increased neutrophil infiltration and mortality rates in patients with AH 72. Additionally, CCL20, a CCR6 ligand involved in chemotaxis of lymphocytes, is positively associated with disease severity in patients with AH. CCL20 is thought to act through chemotactic functions but via induction of pro-inflammatory and pro-fibrotic effects directly on HSCs 73. Elevated chemokine levels in ALD are closely linked to epigenetic reprogramming. In a recent study, researchers performed RNA-seq and ChIP-seq on tissue samples from AH livers and normal livers and found that several CXCL chemokines, including CXCL1, CXCL6, and CXCL8, which are involved in neutrophil recruitment, showed significantly increased expression in the livers of AH patients. Further studies revealed that the promoter regions of the corresponding genes in AH were enriched for active modifications (H3K27ac and H3K4me3) and lacked a repressive marker (H3K27me3). Researchers have demonstrated that the production of CXCL chemokines is regulated by the TNFα/NF-κB signaling pathway. They also identified a super-enhancer containing multiple CXCL loci upstream that promotes TNFα-induced upregulation of CXCL chemokines. Epigenome editing or specific repression of BET proteins, which are transcriptional regulators essential for super-enhancer function, reduces CXCL chemokine expression *in vivo* and *in vitro* and leads to reduced neutrophil infiltration in the AH mouse model. Functional studies on the CXCL super-enhancer confirm its role in inflammatory signaling in AH. Understanding the role of distal cis-regulatory elements and their corresponding epigenetic regulation in AH is critical to gain insight into the disease pathogenesis and may open new avenues for the development of novel mechanistic therapies applicable to other inflammatory diseases in the liver and other organ systems 74.

FK506-binding protein 5 (FKBP5) is a co-chaperone protein known to interact with steroid receptors and is thought to be an important regulator of the stress response 75. Ethanol consumption is associated with FKBP5 in humans, and ethanol has been recently shown to cause reduced levels of DNA methylation in the promoter region of FKBP5, resulting in increased FKBP5 mRNA and protein expression, both in ALD patients and ethanol-fed mice. Activation of TEAD1 leads to increased expression of its novel target CXCL1, which mediates neutrophil recruitment in AH, thereby causing liver inflammation and neutrophil infiltration. Deletion of FKBP5 significantly ameliorates alcohol-induced liver injury, suggesting that it has potential as a therapeutic strategy for ALD 76. Taken together, these findings suggest that regulation of chemokine production through epigenetic pathways may be a potential therapeutic modality for ALD.

#### 2.3.3. Exosomes and extracellular vesicles

Extracellular vesicles are vesicles with a bilayer membrane structure; they are either shed from cell membranes or secreted by cells. These vesicles act as “carriers” for intercellular communication and are capable of secreting and delivering outside the cell various biomolecules, such as proteins, RNA, and DNA. Extracellular vesicles play crucial roles in several biological processes, including immune response, cell growth, differentiation, apoptosis, and tumorigenesis. Among the various types of extracellular vesicles, exosomes are prevalent in immune cells and have several functions such as antigen presentation, immune activation, and immunosuppression. Exosomes contain microRNAs that enter the circulation or are taken up by neighboring cells, allowing miRNAs to modify targets in the recipient cells; thus, they play crucial roles in the development and progression of ALD as regulatory signals, biomarkers, and therapeutic targets 77.

MicroRNAs have various potential functions in the pathogenesis and management of ALD. With respect to pathogenesis, microRNAs act as signaling molecules and can modulate immune cells. For example, monocytes exposed to alcohol can release exosomes carrying miR-27a, which in turn acts as a programming signal to stimulate the polarization of naive monocytes into M2 macrophages 78. Second, microRNAs can serve as disease-specific biomarkers in the diagnosis of ALD. For instance, in blood extracellular vesicles of ASH mice, three miRNAs, namely, let7f, miR-29a, and miR-340, were found to be significantly elevated; no such significant changes in specific miRNA levels were observed in the blood extracellular vesicles of the mouse models of three other conditions involving chronic liver injury—bile duct ligation, NASH, and obesity 79. Further research is needed to identify novel ALD biomarkers and to confirm whether extracellular vesicles containing the abovementioned three miRNAs can serve as reliable biomarkers for the diagnosis of ALD. Finally, microRNAs can be exploited as therapeutic targets to alleviate liver injury caused by alcohol. For instance, miR-122 is a hepatocyte-specific miRNA that shows reduced levels in in human liver and murine hepatocytes on exposure to chronic alcohol intake. This reduction in miR-122 levels promotes liver injury, steatosis, and inflammation, and therapeutic AAV8-mediated miR-122 restoration has been shown to reverse liver injury by inhibiting hypoxia-inducible factor (HIF1α) 80. Therefore, the induction of miR-122 expression shows promise as a therapeutic strategy for ALD and liver fibrosis.

Different microRNAs have varying effects on the inflammatory response in ALD. Certain microRNAs promote an inflammatory response, leading to liver damage. Overexpression of miR-155, miR-154, miR-34c, miR-450a, and miR-204 has recently been shown to increase the production of the inflammatory cytokines TNFα or IL-6 in peripheral blood mononuclear cells after alcohol stimulation, leading to an intensified inflammatory response 43. In contrast, some microRNAs mitigate the inflammatory response and protect the liver from injury. Forkhead box protein O1 (FoxO1) has been identified as a transcription factor for miR-148a, and alcohol exposure causes hepatocyte scorching by inhibiting FoxO1 and reducing miR-148a expression in the hepatocytes, thereby promoting TXNIP overexpression and NLRP3 inflammatory vesicle activation. Overexpression of miR-148a prevents inflammasome activation and cell scorching through TXNIP inhibition 81. Additionally, miR-148a has recently been shown to regulate ADH expression 82. In patients with ALD and animal models of ALD, serum and liver levels of neutrophil-specific miR-233 are elevated, and miR-233 has been shown to be important for the inhibition of neutrophil hyperactivation by suppressing IL-6 production 83. In light of these findings, it is necessary that large-scale clinical cohort studies are conducted to further explore and validate the effectiveness and usefulness of microRNAs in predicting ALD progression, detecting it early, and monitoring treatment response.

### 2.4. Inflammatory pathways

#### 2.4.1. Loss of intestinal integrity

The damaging effects of alcohol abuse on the intestinal mucosa are well-documented. This damage can lead to an increase in intestinal permeability, allowing endotoxins to reach the liver and resulting in an inflammatory response. Tight-junction proteins (TJPs), particularly TJP1, are essential for regulating intestinal permeability, and their expression is epigenetically regulated by inflammation-induced microRNAs. Tang and colleagues have shown that ethanol exposure caused the upregulation of miR-212, which reduced the expression of TJP1 and compromised tight junctions, thus leading to an increase in intestinal permeability in Caco-2 cells. The elevated expression of miR-212 leads to the silencing of TJP1 mRNA, which, in turn, contribute to the progression of ALD. These findings suggest that high miR-212 expression levels are associated with increased intestinal permeability, and therefore, therapeutic regulation of the miR-212-TJP1 axis could block the inflammatory response triggered by alcohol consumption 84. Recent studies 85 have also shown that dysregulation of miR-130a and miR-212 expression in the colonic epithelium can disrupt the epithelial barrier by downregulating PPARγ and OCLN expression. Further investigations are warranted to explore the epigenetic mechanisms involved in the inflammatory pathway.

#### 2.4.2. Aging and inflammation

Aging is associated with various epigenetic shifts that influence gene expression, metabolic pathways, and inflammation relevant to ALD. In a 2017 study 86, researchers noted that hepatic SIRT1 protein expression in middle-aged mice is lower than that in their younger counterparts. The study revealed that SIRT1-induced dip in hepatocytes and HSCs heightened the vulnerability of middle-aged mice to chronic-plus-binge ethanol-induced liver injury. On the other hand, restoration of hepatic SIRT1 protein expression alleviated this injury. Further investigation into how aging-distorted epigenetic status affects ALD 87 revealed some key points: neutrophilic SIRT1 downregulation as part of aging and the alcohol-induced reduction in the expression of the anti-inflammatory factor miR-223, amplifying susceptibility to liver injury. Additionally, aging-related or alcohol-induced downregulation of SIRT1 in neutrophils spurred the acetylation of CCAAT/enhancer-binding protein α (C/EBPα), controlling miR-223 production. Investigations on the exact mechanisms by which aging-related epigenetic changes contribute to ALD are still underway. Research in this area is important to gain an understanding into how aging impacts ALD susceptibility and progression. Such an understanding could facilitate the development of targeted interventions to mitigate the effects of age-related epigenetic changes on liver health.

## 3. Epigenetic alterations in different forms of ALD (Fig. 4)

Alcohol-associated liver disease (ALD) comprises a wide range of hepatic disorders, including asymptomatic steatosis, AH, alcoholic liver fibrosis and cirrhosis, ALD-ACLF and alcohol-related HCC. Different stages of ALD are associated with different epigenetic modifications. In alcoholic steatosis, ethanol treatment downregulates the expression of carnitine palmitoyltransferase-1A (*CPT-1A*) gene and upregulates fatty acid synthase (Fas) by regulating the expression of HDAC. Intracellular adipogenesis in hepatocytes is closely linked to the elevated expression of Fas and decreased expression of CPT-1A. In AH, alcohol consumption causes the downregulation of EHMT2 and SIRT6, while chronic alcohol consumption causes the upregulation of miR-155 and miR-182, thus triggering inflammation. In alcoholic cirrhosis, ethanol induces the activation of KMT2A (histone H3Lys4 methyltransferase), which subsequently mediates histone H3 trimethylation at Lys4 on the elastin gene promoter, leading to chromatin activation and upregulation of elastin and other ECM genes and thereby promoting the progression of liver fibrosis. During the development of liver failure, EZH2 expression significantly increases, and pro-inflammatory cytokines are promoted through the enrichment of H3K27me3 and NF-κB and Akt signaling pathways. In HCC, the expression level of MAT2A is greater than that of MAT1A, leading to lower SAM production and rapid tumor growth.

### 3.1. Alcoholic steatosis

AFLD is primarily marked by increased fat synthesis and reduced fatty acid oxidation in hepatocytes. The development of alcohol-induced hepatic steatosis is closely associated with epigenetic regulatory mechanisms.

Increased histone acetylation is mainly associated with the regulation of hepatic lipid metabolism-related genes, particularly by the action of HDACs. Intracellular adipogenesis in hepatocytes is closely linked to the increased expression of the fatty acid synthase (Fas) and decreased expression of the carnitine palmitoyltransferase-1A (CPT-1A). Acute alcohol intake has been shown to decrease HDAC9 mRNA expression, leading to increased acetylation of the Fas promoter histone and increased chromatin accessibility; this results in transcriptional activation, which promotes hepatic steatosis and adipogenesis 88. Another study found that ethanol treatment downregulates the expression of the *CPT-1A* gene by increasing the CPT-1A promoter region 89 recruitment of HDAC1, which deacetylates the CPT-1A promoter histone H3K9. However, oral administration of tributyrin, a dietary HDAC inhibitor, can inhibit histone deacetylation, thereby reducing the negative effects of ethanol on CPT-1A.

Alcohol-induced steatosis has been linked to the abnormal expression of microRNAs, which regulate inflammation and lipid metabolism through the post-transcriptional modulation of their target mRNAs 90. Studies have identified distinct miRNA expression profiles for alcoholic and non-alcoholic fatty liver, with some experiments on mouse models showing that the Lieber-DeCarli alcohol diet induced the upregulation of 1% of miRNAs and the downregulation of 1%, while the methionine-choline-deficient (MCD) diet induced the upregulation of 3% and downregulation of 1% 91. Interestingly, both these diets induced the upregulation of miR-705 and miR-1224; the MCD diet induced the upregulation of miR-199a-3p, miR-182, and miR-183, while the Lieber-DeCarli diet induced their downregulation. These findings together indicate that abnormal miRNA expression is a feature common to both alcoholic and non-alcoholic fatty liver. A recent study on rat models of AFLD revealed that miR-181b-5p and PRMT1 expression was increased, while PIAS1 expression was decreased in liver tissue 92. Further analysis showed that miR-181b-5p promoted PRMT1 expression by inhibiting *PIAS1*, which was identified as a target gene of miR-181b-5p. Notably, overexpression of PIAS1 or inhibition of PRMT1 by the inhibition of miR-181b-5p caused improvement of steatosis in rats with AFLD. These results suggest that regulating miRNA expression patterns may hold promise as a therapeutic strategy for alcohol-related fatty liver disease.

Alcohol-induced hepatic steatosis has also been linked to DNA methylation, with PCSK9 being a key regulator of the low-density lipoprotein cholesterol (LDL-C) primarily expressed in the liver. Alcohol exposure has also been shown to cause variant methylation at the promoter region of PCSK9, which can affect its expression and contribute to the regulation of LDL-C levels 93. Therefore, basic and clinical research is warranted to further investigate potential epigenetic mechanisms that can be targeted for the prevention and treatment of alcohol-induced hepatic steatosis.

### 3.2. AH

#### 3.2.1. Mild AH

Both histone modifications and miRNAs, by virtue of their crucial roles in regulating the inflammatory response in mild AH, represent potential therapeutic targets. Downregulation of EYIHMT2 (G9a), an H3K9 methyltransferase, has been observed in mouse models of AH, resulting in endoplasmic reticulum stress and the upregulation of several genes associated with oxidative stress signaling, including HSPA5, ATF4, DDIT3, CASP12, and SREBF1. Therefore, enhancing the EHMT2 expression to alleviate endoplasmic reticulum stress may help reduce the degree of liver inflammation in ALD 94. SIRT6, an NAD^+^-dependent histone deacetylase, has been shown to be downregulated in both mouse models of ALD mice and patients with AH. However, hepatic SIRT6 overexpression in mice has been found to reverse ethanol-induced damage by enhancing transcriptional the induction of Mt1 and Mt2 genes and improving the hepatic defense system against alcohol toxicity 95. Additionally, a recent study demonstrated that chronic alcohol feeding in mice upregulates miR-155 expression in the liver macrophages and promoting inflammatory responses through the activation of TLR4 signaling, thereby triggering inflammation. Knockdown of miR-155 alleviated LPS/TLR4-induced inflammation in AH 39.

#### 3.2.2. SAH

Severe alcoholic hepatitis (SAH) is characterized by the coexistence of systemic inflammation and immune dysfunction in the form of impaired immune cell responses to pathogens and their products, thereby leading to an increased susceptibility to infection. A recent study used single-cell RNA sequencing to determine the composition and proportion of immune cells in patients with early AH, severe AH, HCV, HCV combined with cirrhosis, and NAFLD. The study revealed that patients with SAH had the most extensive alteration in immune cell composition 96. Another study showed a sustained decrease in the proportion of monocytes expressing IL-6, IL-1β, TNF-α, and IL-12/23p4 cytokines in patients with SAH. The investigators also analyzed the epigenomic landscape of these monocytes, and found that CD14^+^ monocytes from patients with SAH exhibited altered transcriptional and epigenomic profiles 97. In AH patients, the expression profile of miRNAs is known to be substantially altered. Unlike the case with other liver diseases, in AH, miR-182 was the most highly expressed miRNA and its levels correlated with the degree of catheter response, disease severity, and short-term mortality 98. Furthermore, the progression of AH to ACLF is influenced by epigenetic modifications, and the development of hepatocellular failure in AH patients is characterized by a significant reduction in the hepatocyte nuclear factor 4 alpha (HNF4α)-dependent gene expression. This reduction can be attributed to the highly methylated HNF4α-dependent genes in patients with AH. These study results suggest that epigenetic drivers that regulate HNF4α-dependent gene expression may be beneficial in AH patients 99.

### 3.3. Alcoholic liver fibrosis and cirrhosis

Cirrhosis is a progressive liver disease that is considered systemic because it affects various organs and organ systems, including the immune system 100. A recent study using immunofluorescence analysis found that the expression of M1 and M2 macrophages is increased in patients with alcoholic liver cirrhosis and is associated with the promotion of fibrosis 4. Chronic liver injury leads to liver inflammation and fibrosis, where HSCs transdifferentiate into myofibroblasts, resulting in the secretion of ECM proteins and consequent fibrous scarring 101. The transdifferentiation of HSCs is partially mediated by DNA methylation 102, and in a mouse model, treatment of HSCs with the DNMT inhibitor 5-azadC was shown to prevent HSC transdifferentiation 103. In a recent study, Page and his colleagues treated HSCs with ethanol at the transdifferentiation stage and found that ethanol-induced activation of KMT2A (histone H3Lys4 methyltransferase), which then mediated histone H3 trimethylation at Lys4 on the elastin gene promoter; this leads to chromatin activation and upregulation of elastin and other ECM genes 104. This study suggests that alcohol-induced histone trimethylation triggers the upregulation of genes in HSCs, thereby promoting liver fibrosis. Therefore, specific interventions targeting alcohol-induced Lys4 histone trimethylation or fibrosis gene expression could be employed as preventive measure against alcoholic liver fibrosis.

### 3.4. ALD-ACLF

ACLF is characterized by a high degree of systemic inflammation, an exaggerated compensatory anti-inflammatory response, and a failure and dysregulation of immune cells. These changes ultimately lead to multi-organ failure and contribute to a high short-term mortality 105. Recent studies have shown that the most common cause of ACLF is alcohol consumption, followed by infection 106. The pathogenesis of liver failure is characterized by a significant increase in EZH2 expression and promotion of pro-inflammatory cytokines through the enrichment of H3K27me3 and NF-κB and Akt signaling pathways. The EZH2 inhibitor GSK126 has been shown to ameliorate liver injury and improve survival in mice with liver failure by inhibiting TNF and other indispensable pro-inflammatory cytokines. These findings together suggest that EZH2/H3K27me3 plays a role in liver failure by regulating immune activation, as evidenced by the fact that GSK126-induced inhibition of EZH2-catalyzed H3K27me3 can significantly attenuate liver injury and reduce inflammatory cell infiltration. This may be partially responsible for the reduction in the levels of pro-inflammatory cytokines. These findings provide compelling evidence for the potential clinical application of EZH2 methyltransferase targeting in the treatment of liver failure 37.

### 3.5. ALD-HCC

HCC accounts for over 80% of primary liver cancers worldwide and is the leading cause of cancer-related deaths in many regions across the world 107. Chronic alcohol consumption is a well-known independent risk factor for the development of HCC. Lambert and colleagues conducted a study on DNA methylation of HCC-associated genes and discovered that the presence/absence of aberrant DNA methylation patterns helped distinguished between HCC secondary to hepatitis B virus (HBV) and HCV infection and HCC associated with alcohol consumption. In specific terms, the levels of the O6-methylguanine-DNA methyltransferase methylation were significantly higher in alcohol-associated HCC than that in HBV/HCV-associated HCC, which suggests that alcohol may cause targeted hypermethylation of specific genes. However, the alcohol-induced increase in MGMT methylation appears to contradict its decrease during HCC progression. This discrepancy could be because the lower levels of MGMT methylation found in most HCC samples outweigh the relatively small proportion of increase in MGMT hypermethylation occurring in alcohol-associated HCC 108. For further clarity, studies are warranted to determine whether alcohol intake affects MGMT directly or indirectly via its metabolites. Methylated forms of the associated genes could then serve as biomarkers for malignant transformation and risk assessment.

The methionine adenosyltransferase. (MAT) family comprises three members: MAT1A, MAT2A, and MAT2B. While MAT1A synthesizes SAM only in normal liver tissues and bile duct epithelial cells, MAT2A is responsible for SAM production in both extrahepatic normal and cancerous tissues; MAT2B does not exhibit any catalytic activity. Notably, in HCC, the expression of MAT2A is more dominant than that of MAT1A, thereby leading to decreased SAM production and rapid tumor growth. In a study conducted by Lu and colleagues, rats were fed ethanol for 9 weeks, which resulted in a switch of predominance from MAT1A to MAT2A, leading to reduced SAM production, hypomethylation of MYC, transcriptional upregulation, DNA strand breaks, and liver damage 109. Recent studies have also suggested that the MAT1A:MAT2A expression ratio could be a potential biomarker for predicting the progression and prognosis of human HCC: a higher MAT1A:MAT2A ratio has been shown to be negatively correlated with cell proliferation and genomic instability and positively correlated with apoptosis and global DNA methylation 110.

Alterations in the expression and mutations of epigenetic modifiers are known to be prevalent in human HCC. In particular, HCC tissue shows an upregulation of 43% of the 90 evaluated epigenetic modifiers, as compared to normal human tissue. Additionally, high expression levels of 12 of these epigenetic modifiers were associated with the worst prognosis among HCC patients. Human HCC is characterized by the dysregulation of several histone post-translational modifications (HPTMs), writers (HATS and KMTs), readers (BRDs), erasers (KDMs), and somatic mutations. Given the critical role of HPTMs in HCC development, growth, and metastasis, pharmacological inhibition of these epigenetic pathways may represent a novel therapeutic strategy. Two histone deacetylase (HDAC) inhibitors, belinostat and reminostat, have been recently investigated in phase I/II clinical trials in HCC patients, which have shown potential efficacy of these targeted therapies 111. To sum up, the detection of promoter methylation of specific genes may help identify cells with potential for malignant transformation in precancerous (cirrhotic) lesions.

## Limitation and future studies

The field of epigenetic drug therapy is currently facing significant challenges. First, since epigenetic modifications are pervasive across almost all genes in the body, the lack of target specificity and sensitivity of the currently available drugs is a major concern. Due to these limitations, these drugs can cause extensive and difficult-to-control epigenetic modifications, thereby compromising their efficacy. To address this concern, future efforts towards drug development in this field should be focused on optimizing the sensitivity and specificity of epigenetic drugs. Second, given the significant inter-individual variability in epigenetic modifications, there are likely to be individual differences in gene expression patterns and clinical disease phenotypes. This makes it imperative that the therapeutic agent is tailored to the individual, which may negatively affect the widespread application of epigenetic drugs. Finally, the sites of each type of modification vary significantly, since epigenetic modifications can occur in a wide range of locations, including the promoter and coding regions of genes, as well as upstream and downstream participants such as enhancers. Identifying the specific sites and levels of the modifications is necessary to reduce the toxic effects of drug use. Furthermore, future studies should explore the impact of different types of alcohol, such as liquor and beer, as well as different drinking patterns on epigenetic modifications in ALD. It is also essential to investigate the role of epigenetic modifications on specific immune cells by experimentation with the knockdown of immune cell-specific epigenetics-modifying enzymes. Overall, we expect epigenetics to play a central role in ALD research over the next decade, with a greater emphasis on characterizing the interplay between epigenetic modifications and the immune microenvironment.

## Conclusion

The incidence of ALD is progressively escalating, paralleled by a rise in the associated morbidity and mortality rates. Recent investigations have revealed that alterations in the immune microenvironment play crucial roles in the pathogenesis of ALD. In this context, epigenetic modifications within the immune system represent a potential target for addressing alcohol-induced diseases. In this paper, we meticulously elaborate on the relationship between alcohol consumption and epigenetics, focusing specifically on the impact of alcohol metabolites (such as ethanol, acetaldehyde, and acetic acid) and enzymes (ADH, ALDH2, and CYP2E1) on epigenetic modifications. Given that methionine metabolism produces SAM, the donor for all methylation reactions *in vivo*, and alcohol consumption is closely related to folate, vitamin B6, and vitamin B12, which are required for methionine metabolism, we further summarize the relationship between alcohol consumption and methionine metabolism to gain insight into the mechanisms underlying the role of alcohol in the regulation of epigenetic modifications. Additionally, we also elaborate on the effect of alcohol consumption on epigenetic modulation of the immune microenvironment, including its impact on immune cells, immune-related cells, immune mediators, and inflammatory pathways. Finally, we innovatively summarize the differences between the epigenetic modifications in each disease stage, with a view to improving the diagnosis, monitoring, treatment, and prognosis of the disease in clinical practice.

## Figures and Tables

**Figure 1:**
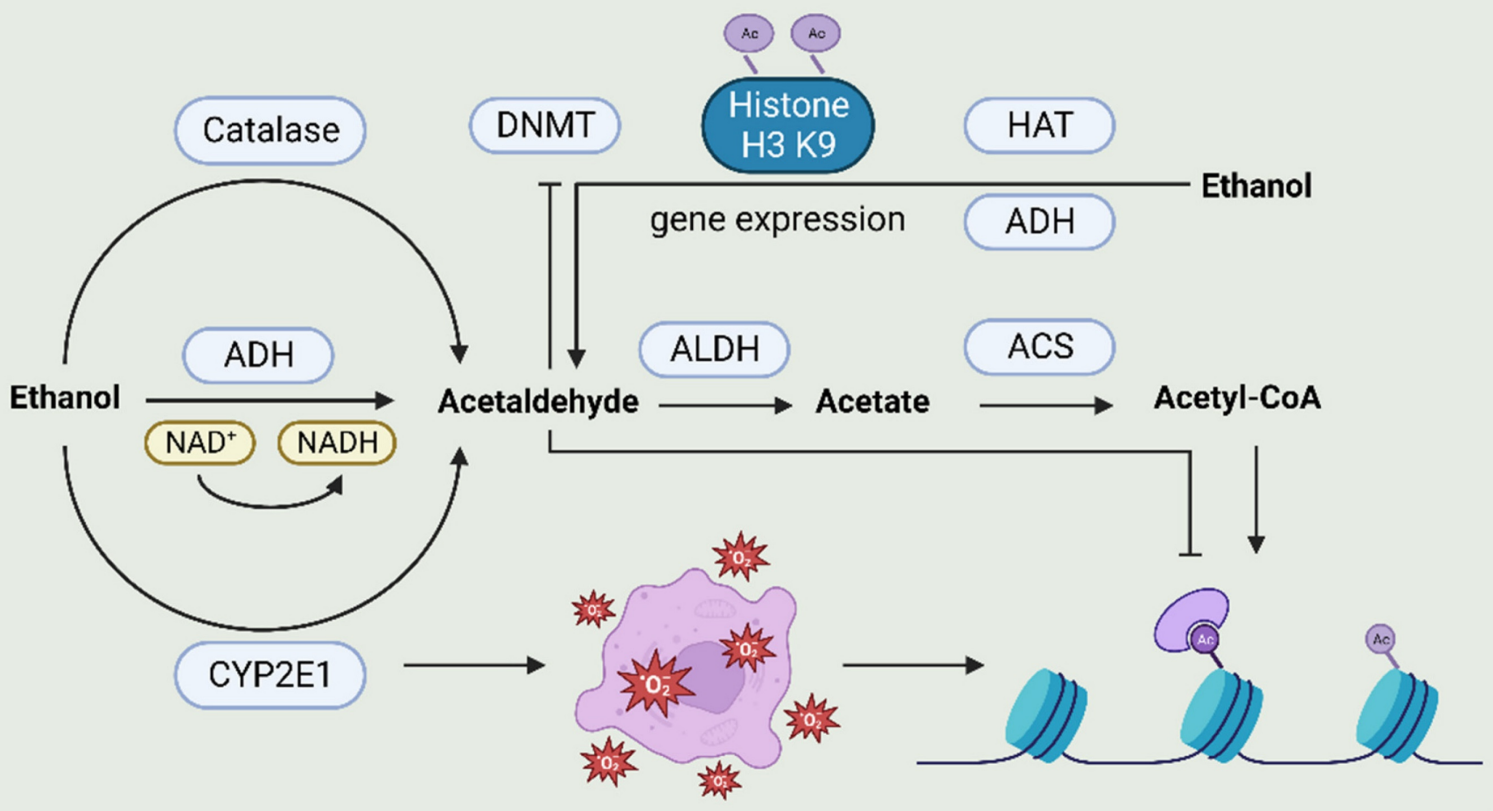
**The relationship between alcohol metabolism and epigenetics.** After alcohol consumption, it is primarily converted to acetaldehyde through the action of alcohol dehydrogenase (ADH), cytochrome P450 2E1 (CYP2E1), and catalase. Subsequently, acetaldehyde is further converted to acetic acid by acetaldehyde dehydrogenase (ALDH). Acetic acid can be synthesized into acetyl-CoA by acetyl-CoA synthetase (ACS), and acetyl-CoA serves as a substrate for histone acetylation. Additionally, CYP2E1 can generate reactive oxygen species (ROS). ROS is linked to histone H3 acetylation at Lys9.Acetaldehyde inhibits DNA methyltransferase activity, ethanol increases the acetylation of H3-Lys9 by regulating HAT and that histone acetylation may be the basis for ethanol-induced ADHI gene expression. ACS, acetyl-CoA synthetase; ADH, alcohol dehydrogenase; ALDH, acetaldehyde dehydrogenase; CYP2E1, cytochrome P450 2E1; DNMT, DNA methyltransferase; HAT, histone acetyltransferase; ROS, reactive oxygen species.

**Figure 3:**
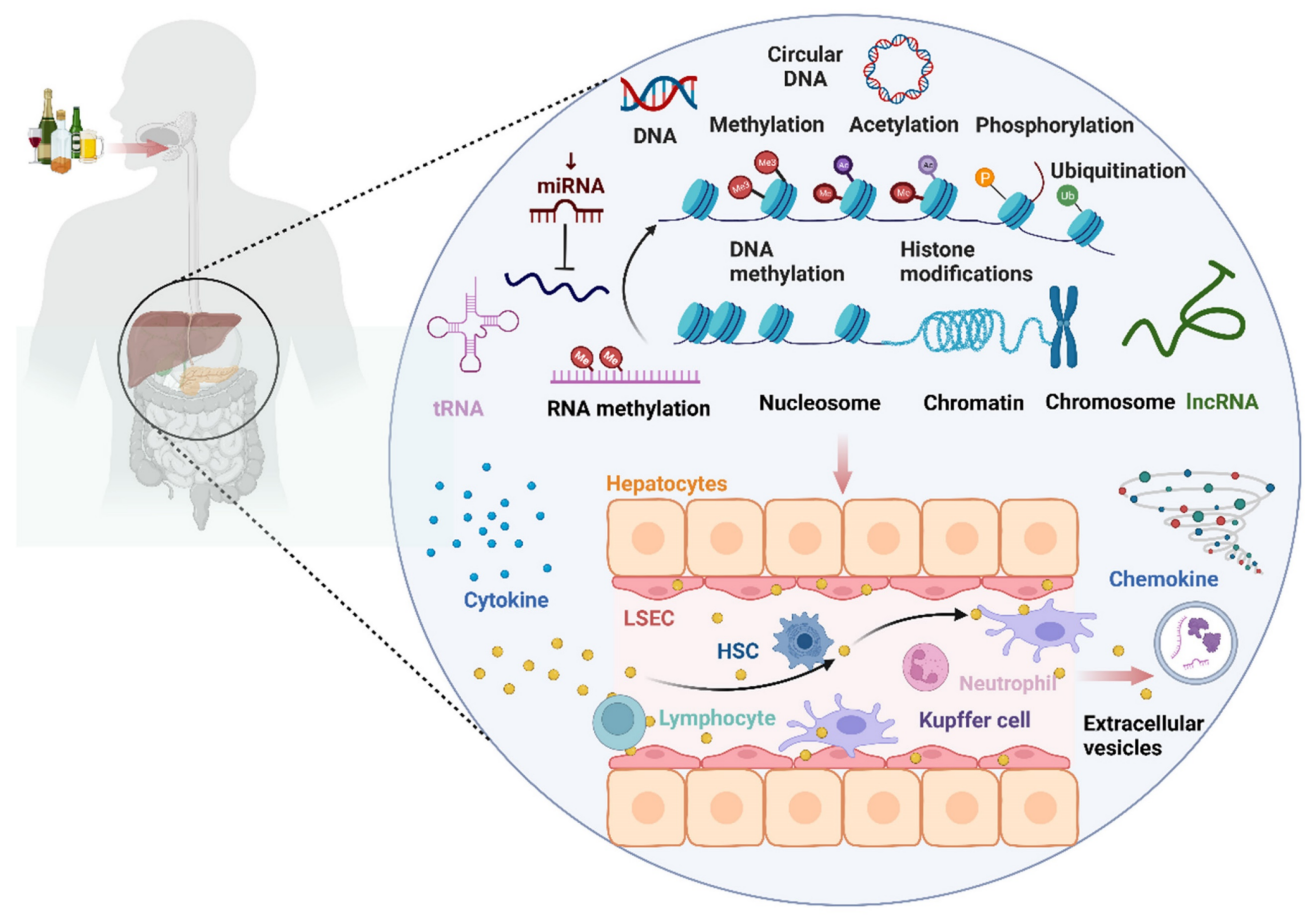
** The landscape of epigenetic regulatory network of the immune microenvironment in ALD.** Alcohol consumption can impact the occurrence and progression of alcohol-associated liver disease (ALD) by modulating immune cells and immune-related cells such as macrophages, neutrophils, hepatic stellate cell (HSCs), T lymphocytes, natural killer T (NKT) cells, and liver sinusoidal endothelial cells (LSECs) through epigenetic modifications (DNA methylation, histone modifications, microRNA, RNA methylation). This, in turn, influences the levels of certain cytokines, chemokines, and extracellular vesicles (EVs) involved in ALD.

**Figure 4:**
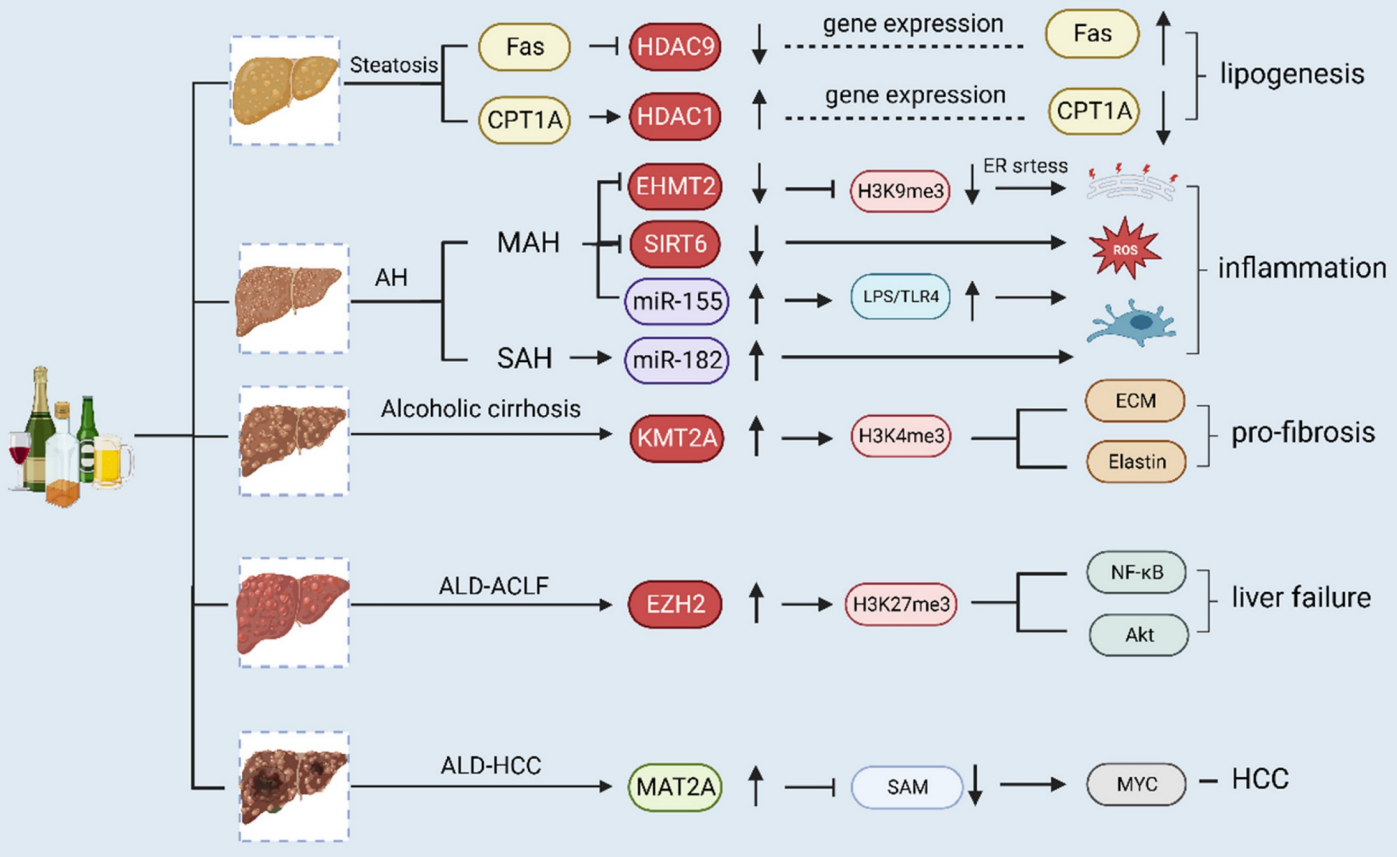
** Different epigenetic modifications in different forms of alcoholic liver disease.** Alcohol- associated liver disease (ALD) comprises a wide range of hepatic disorders, including asymptomatic steatosis, alcoholic hepatitis (AH), alcoholic liver fibrosis and cirrhosis, ALD-Acute-on-chronic liver failure (ACLF) and alcohol-related hepatocellular carcinoma (HCC). Different stages of ALD have different epigenetic modifications. In alcoholic steatosis, ethanol treatment downregulates carnitine palmitoyltransferase-1A (CPT-1A) gene expression and upregulates fatty acid synthase (Fas) by regulating HDAC. Intracellular adipogenesis in hepatocytes is closely linked to the elevated expression of the Fas and decreased expression of the CPT-1A.In AH, alcohol consumption downregulated EHMT2 and SIRT6, chronic alcohol consumption upregulates miR-155 and miR-182, triggering inflammation. In alcoholic cirrhosis, ethanol-induced activation of KMT2A (histone H3Lys4 methyltransferase), which subsequently mediated histone H3 trimethylation at Lys4 on the elastin gene promoter, leading to chromatin activation and upregulation of elastin and other ECM genes, promoting the progression of liver fibrosis. During the development of liver failure, EZH2 expression significantly increases, and pro-inflammatory cytokines are promoted through the enrichment of H3K27me3 and NF-κB and Akt signaling pathways. In HCC, the expression of MAT2A is more dominant than that of MAT1A, leading to lower SAM production and rapid tumor growth. ACLF, acute-on-chronic liver failure; AH, alcoholic hepatitis; ALD, alcohol-associated liver disease; CPT-1A, carnitine palmitoyltransferase-1A; ECM, extracellular matrix; EHMT2 euchromatic Histone-Lysine N-Methyltransferase 2; ER, endoplasmic reticulum; EZH2, enhancer of Zeste Homolog 2; Fas fatty acid synthase; HCC, hepatocellular carcinoma; HDAC, histone deacetylase; KAT2A, lysine acetyltransferase 2A; LPS, lipopolysaccharide; MAH, mild alcoholic hepatitis; MAT, methionine adenosyltransferase.; NF-κB, nuclear transcription factor-κB; ROS, reactive oxygen species; SAH, severe alcoholic hepatitis; SIRT6, sirtuins 6; TLR4, toll-like receptor 4

## Abbreviations

ACLF: acute-on-chronic liver failure
ACS: acetyl-CoA synthetase
ADH: alcohol dehydrogenase
AFLD: alcoholic fatty liver disease
AH: alcoholic hepatitis
ALD: alcohol- associated liver disease
ALDH: acetaldehyde dehydrogenase
AUD: alcohol use disorder
BRD: bromodomain
Brg1: brahma-related gene 1
CaN: calcium-regulated neurophosphatase
ChIP: chromatin immunoprecipitation
CPT-1A: carnitine palmitoyltransferase-1A
CYP2E1: cytochrome P450 2E1
DNMT: DNA methyltransferase
ECM: extracellular matrix
EHMT2: euchromatic Histone-Lysine N-Methyltransferase 2
EZH2: enhancer of Zeste Homolog 2
Fas: fatty acid synthase
FKBP5: FK506-binding protein 5
FoxO1: forkhead box protein O1
FOXP3: forkhead Box P3
GSH: glutathione
HAT: histone acetyltransferase
HCC: hepatocellular carcinoma
HCV: hepatitis C virus
HDAC: histone deacetylase
HDNs: high-density neutrophils
HIF1α: hypoxia-inducible factor1α
HNF4α: hepatocyte nuclear factor 4 alpha
HSC: hepatic stellate cell
ILC1: innate lymphoid cells 1
JMJD3: jumonji domain-containing protein 3
KAT2A: lysine Acetyltransferase 2A
KDM: lysine demethylase
KMT: lysine methyltransferase
LDL-C: low-density lipoprotein cholesterol
LDNs: low-density neutrophils
LSEC: liver sinusoidal endothelial cell
MAT: methionine adenosyltransferase
MCD: methionine-choline-deficient diet
MCP-1: monocyte chemoattractant protein-1
MGMT: O-6-Methylguanine-DNA Methyltransferase
NASH: non-alcoholic steatohepatitis
Nets: neutrophil extracellular traps
NF-κB: nuclear transcription factor-κB
OCLN: occludin
PCSK9: proprotein convertase subtilisin/kexin type 9
PIAS1: protein inhibitor of activated STAT 1
PPAR: peroxisome proliferator-activated receptor
PRMT1: protein arginine methyltransferase 1
PSTPIP2: proline-serine-threonine phosphatase interacting protein 2
RCAN1: regulator of calcium-regulated neurophosphatase 1
ROS: reactive oxygen species
SAH: S-adenosylhomocysteine
SAH: severe alcoholic hepatitis
SAM: S-adenosylmethionine
SIRT6: sirtuins 6
SOCS1: suppressor of cytokine signaling 1
STAT-1: signal transducer and activator of transcription-1
TCRs: T-cell receptors
TEAD1: TEA domain transcription factor
TLR4: toll-like receptor 4
TRAF2: tumor necrosis factor receptor-associated factor 2
Treg: regulatory T cell
TXNIP: thioredoxin-interacting protein
ZSWIM3: zinc-finger swim-type containing 3

## References

[1] Singal AK, Mathurin P (2021). Diagnosis and Treatment of Alcohol-Associated Liver Disease: A Review. Jama.

[2] Huang DQ, Mathurin P, Cortez-Pinto H, Loomba R (2023). Global epidemiology of alcohol-associated cirrhosis and HCC: trends, projections and risk factors. Nat Rev Gastroenterol Hepatol.

[3] White AM, Castle IP, Powell PA, Hingson RW, Koob GF (2022). Alcohol-Related Deaths During the COVID-19 Pandemic. Jama.

[4] Ren A, He W, Rao J, Ye D, Cheng P, Jian Q (2023). Dysregulation of innate cell types in the hepatic immune microenvironment of alcoholic liver cirrhosis. Front Immunol.

[5] Cho Y, Bukong TN, Tornai D, Babuta M, Vlachos IS, Kanata E (2023). Neutrophil extracellular traps contribute to liver damage and increase defective low-density neutrophils in alcohol-associated hepatitis. J Hepatol.

[6] Park PH, Miller R, Shukla SD (2003). Acetylation of histone H3 at lysine 9 by ethanol in rat hepatocytes. Biochem Biophys Res Commun.

[7] Park PH, Lim RW, Shukla SD (2005). Involvement of histone acetyltransferase (HAT) in ethanol-induced acetylation of histone H3 in hepatocytes: potential mechanism for gene expression. Am J Physiol Gastrointest Liver Physiol.

[8] Park PH, Lim RW, Shukla SD (2012). Gene-selective histone H3 acetylation in the absence of increase in global histone acetylation in liver of rats chronically fed alcohol. Alcohol Alcohol.

[9] Vijayraghavan S, Porcher L, Mieczkowski PA, Saini N (2022). Acetaldehyde makes a distinct mutation signature in single-stranded DNA. Nucleic Acids Res.

[10] Garro AJ, McBeth DL, Lima V, Lieber CS (1991). Ethanol consumption inhibits fetal DNA methylation in mice: implications for the fetal alcohol syndrome. Alcohol Clin Exp Res.

[11] Ganesan M, Zhang J, Bronich T, Poluektova LI, Donohue TM Jr, Tuma DJ (2015). Acetaldehyde accelerates HCV-induced impairment of innate immunity by suppressing methylation reactions in liver cells. Am J Physiol Gastrointest Liver Physiol.

[12] Shvedunova M, Akhtar A (2022). Modulation of cellular processes by histone and non-histone protein acetylation. Nat Rev Mol Cell Biol.

[13] Kriss CL, Gregory-Lott E, Storey AJ, Tackett AJ, Wahls WP, Stevens SM Jr (2018). *In vivo* Metabolic Tracing Demonstrates the Site-Specific Contribution of Hepatic Ethanol Metabolism to Histone Acetylation. Alcohol Clin Exp Res.

[14] Wu X, Fan X, Miyata T, Kim A, Cajigas-Du Ross CK, Ray S (2023). Recent Advances in Understanding of Pathogenesis of Alcohol-Associated Liver Disease. Annu Rev Pathol.

[15] Dannenberg LO, Chen HJ, Tian H, Edenberg HJ (2006). Differential regulation of the alcohol dehydrogenase 1B (ADH1B) and ADH1C genes by DNA methylation and histone deacetylation. Alcohol Clin Exp Res.

[16] Seo W, Gao Y, He Y, Sun J, Xu H, Feng D (2019). ALDH2 deficiency promotes alcohol-associated liver cancer by activating oncogenic pathways via oxidized DNA-enriched extracellular vesicles. J Hepatol.

[17] Chen X, Luo J, Gao S, Jiang J, Yang B, Zhang Z (2022). miR-671-5p Promotes Cell Proliferation, Invasion, and Migration in Hepatocellular Carcinoma through Targeting ALDH2. Crit Rev Eukaryot Gene Expr.

[18] Shankarappa B, Mahadevan J, Murthy P, Purushottam M, Viswanath B, Jain S (2023). Hypomethylation of Long Interspersed Nucleotide Elements and Aldehyde Dehydrogenase in Patients of Alcohol Use Disorder with Cirrhosis. DNA Cell Biol.

[19] Liu L, Yang X, Zhao F, Gao C, Zhang N, Bao J (2021). Hypermethylation of the OPRM1 and ALDH2 promoter regions in Chinese Han males with alcohol use disorder in Yunnan Province. Am J Drug Alcohol Abuse.

[20] Xue L, Yang F, Han Z, Cui S, Dai S, Xu F (2018). ALDH2 mediates the dose-response protection of chronic ethanol against endothelial senescence through SIRT1/p53 pathway. Biochem Biophys Res Commun.

[21] Choudhury M, Park PH, Jackson D, Shukla SD (2010). Evidence for the role of oxidative stress in the acetylation of histone H3 by ethanol in rat hepatocytes. Alcohol.

[22] Grove TL, Benner JS, Radle MI, Ahlum JH, Landgraf BJ, Krebs C (2011). A radically different mechanism for S-adenosylmethionine-dependent methyltransferases. Science.

[23] Lu SC, Huang ZZ, Yang H, Mato JM, Avila MA, Tsukamoto H (2000). Changes in methionine adenosyltransferase and S-adenosylmethionine homeostasis in alcoholic rat liver. Am J Physiol Gastrointest Liver Physiol.

[24] Kancherla V, Botto LD, Rowe LA, Shlobin NA, Caceres A, Arynchyna-Smith A (2022). Preventing birth defects, saving lives, and promoting health equity: an urgent call to action for universal mandatory food fortification with folic acid. Lancet Glob Health.

[25] Medici V, Halsted CH (2013). Folate, alcohol, and liver disease. Mol Nutr Food Res.

[26] Zhao H, Guo P, Zuo Y, Wang Y, Zhao H, Lan T (2022). Folic acid intervention changes liver Foxp3 methylation and ameliorates the damage caused by Th17/Treg imbalance after long-term alcohol exposure. Food Funct.

[27] Halsted CH (2013). B-Vitamin dependent methionine metabolism and alcoholic liver disease. Clin Chem Lab Med.

[28] Labadarios D, Rossouw JE, McConnell JB, Davis M, Williams R (1977). Vitamin B6 deficiency in chronic liver disease-evidence for increased degradation of pyridoxal-5'-phosphate. Gut.

[29] Lindenbaum J, Lieber CS (1969). Alcohol-induced malabsorption of vitamin B12 in man. Nature.

[30] Kanazawa S, Herbert V (1985). Total corrinoid, cobalamin (vitamin B12), and cobalamin analogue levels may be normal in serum despite cobalamin in liver depletion in patients with alcoholism. Lab Invest.

[31] Tripathi M, Singh BK, Zhou J, Tikno K, Widjaja A, Sandireddy R (2022). Vitamin B(12) and folate decrease inflammation and fibrosis in NASH by preventing syntaxin 17 homocysteinylation. J Hepatol.

[32] Feng D, Xiang X, Guan Y, Guillot A, Lu H, Chang C (2023). Monocyte-derived macrophages orchestrate multiple cell-type interactions to repair necrotic liver lesions in disease models. J Clin Invest.

[33] Maccioni L, Kasavuli J, Leclercq S, Pirlot B, Laloux G, Horsmans Y (2023). Toll-like receptor 2 activation in monocytes contributes to systemic inflammation and alcohol-associated liver disease in humans. Hepatol Commun.

[34] Xu JJ, Zhu L, Li HD, Du XS, Li JJ, Yin NN (2022). DNMT3a-mediated methylation of PSTPIP2 enhances inflammation in alcohol-induced liver injury via regulating STAT1 and NF-κB pathway. Pharmacol Res.

[35] Li HD, Chen X, Xu JJ, Du XS, Yang Y, Li JJ (2020). DNMT3b-mediated methylation of ZSWIM3 enhances inflammation in alcohol-induced liver injury via regulating TRAF2-mediated NF-κB pathway. Clin Sci (Lond).

[36] Sureshchandra S, Stull C, Ligh BJK, Nguyen SB, Grant KA, Messaoudi I (2019). Chronic heavy drinking drives distinct transcriptional and epigenetic changes in splenic macrophages. EBioMedicine.

[37] Zhou T, Sun Y, Li M, Ding Y, Yin R, Li Z (2018). Enhancer of zeste homolog 2-catalysed H3K27 trimethylation plays a key role in acute-on-chronic liver failure via TNF-mediated pathway. Cell Death Dis.

[38] Villagra A, Cheng F, Wang HW, Suarez I, Glozak M, Maurin M (2009). The histone deacetylase HDAC11 regulates the expression of interleukin 10 and immune tolerance. Nat Immunol.

[39] Bala S, Csak T, Kodys K, Catalano D, Ambade A, Furi I (2017). Alcohol-induced miR-155 and HDAC11 inhibit negative regulators of the TLR4 pathway and lead to increased LPS responsiveness of Kupffer cells in alcoholic liver disease. J Leukoc Biol.

[40] Hartmann P, Tacke F (2016). Tiny RNA with great effects: miR-155 in alcoholic liver disease. J Hepatol.

[41] Norkina O, Dolganiuc A, Catalano D, Kodys K, Mandrekar P, Syed A (2008). Acute alcohol intake induces SOCS1 and SOCS3 and inhibits cytokine-induced STAT1 and STAT3 signaling in human monocytes. Alcohol Clin Exp Res.

[42] Bala S, Csak T, Saha B, Zatsiorsky J, Kodys K, Catalano D (2016). The pro-inflammatory effects of miR-155 promote liver fibrosis and alcohol-induced steatohepatitis. J Hepatol.

[43] Lewis SA, Doratt B, Sureshchandra S, Pan T, Gonzales SW, Shen W (2022). Profiling of extracellular vesicle-bound miRNA to identify candidate biomarkers of chronic alcohol drinking in nonhuman primates. Alcohol Clin Exp Res.

[44] Hsu SH, Wang B, Kota J, Yu J, Costinean S, Kutay H (2012). Essential metabolic, anti-inflammatory, and anti-tumorigenic functions of miR-122 in liver. J Clin Invest.

[45] Momen-Heravi F, Saha B, Kodys K, Catalano D, Satishchandran A, Szabo G (2015). Increased number of circulating exosomes and their microRNA cargos are potential novel biomarkers in alcoholic hepatitis. J Transl Med.

[46] Wan Y, Slevin E, Koyama S, Huang CK, Shetty AK, Li X (2023). miR-34a regulates macrophage-associated inflammation and angiogenesis in alcohol-induced liver injury. Hepatol Commun.

[47] Pan XS, Li BW, Wang LL, Li N, Lin HM, Zhang J (2023). Kupffer cell pyroptosis mediated by METTL3 contributes to the progression of alcoholic steatohepatitis. Faseb j.

[48] Nathan C (2006). Neutrophils and immunity: challenges and opportunities. Nat Rev Immunol.

[49] Fischer J, Walter C, Tönges A, Aleth H, Jordão MJC, Leddin M (2019). Safeguard function of PU.1 shapes the inflammatory epigenome of neutrophils. Nat Immunol.

[50] Li N, Liu H, Xue Y, Xu Z, Miao X, Guo Y (2023). Targetable Brg1-CXCL14 axis contributes to alcoholic liver injury by driving neutrophil trafficking. EMBO Mol Med.

[51] Ma J, Guillot A, Yang Z, Mackowiak B, Hwang S, Park O (2022). Distinct histopathological phenotypes of severe alcoholic hepatitis suggest different mechanisms driving liver injury and failure. J Clin Invest.

[52] Cheng C, Zhang Q, Li Y, Jiang J, Xie L, Shen H (2023). Interplay Between Liver Type 1 Innate Lymphoid Cells and NK Cells Drives the Development of Alcoholic Steatohepatitis. Cell Mol Gastroenterol Hepatol.

[53] Sinclair LV, Howden AJ, Brenes A, Spinelli L, Hukelmann JL, Macintyre AN (2019). Antigen receptor control of methionine metabolism in T cells. Elife.

[54] Roy DG, Chen J, Mamane V, Ma EH, Muhire BM, Sheldon RD (2020). Methionine Metabolism Shapes T Helper Cell Responses through Regulation of Epigenetic Reprogramming. Cell Metab.

[55] Ramos GP, Bamidele AO, Klatt EE, Sagstetter MR, Kurdi AT, Hamdan FH (2023). G9a Modulates Lipid Metabolism in CD4 T Cells to Regulate Intestinal Inflammation. Gastroenterology.

[56] Pan XY, You HM, Wang L, Bi YH, Yang Y, Meng HW (2019). Methylation of RCAN1.4 mediated by DNMT1 and DNMT3b enhances hepatic stellate cell activation and liver fibrogenesis through Calcineurin/NFAT3 signaling. Theranostics.

[57] Perugorria MJ, Wilson CL, Zeybel M, Walsh M, Amin S, Robinson S (2012). Histone methyltransferase ASH1 orchestrates fibrogenic gene transcription during myofibroblast transdifferentiation. Hepatology.

[58] Jiang Y, Xiang C, Zhong F, Zhang Y, Wang L, Zhao Y (2021). Histone H3K27 methyltransferase EZH2 and demethylase JMJD3 regulate hepatic stellate cells activation and liver fibrosis. Theranostics.

[59] Barcena-Varela M, Paish H, Alvarez L, Uriarte I, Latasa MU, Santamaria E (2021). Epigenetic mechanisms and metabolic reprogramming in fibrogenesis: dual targeting of G9a and DNMT1 for the inhibition of liver fibrosis. Gut.

[60] Sørensen KK, Simon-Santamaria J, McCuskey RS, Smedsrød B (2015). Liver Sinusoidal Endothelial Cells. Compr Physiol.

[61] Yang Y, Sangwung P, Kondo R, Jung Y, McConnell MJ, Jeong J (2021). Alcohol-induced Hsp90 acetylation is a novel driver of liver sinusoidal endothelial dysfunction and alcohol-related liver disease. J Hepatol.

[62] Tateya S, Rizzo NO, Handa P, Cheng AM, Morgan-Stevenson V, Daum G (2011). Endothelial NO/cGMP/VASP signaling attenuates Kupffer cell activation and hepatic insulin resistance induced by high-fat feeding. Diabetes.

[63] Adams C, Conigrave JH, Lewohl J, Haber P, Morley KC (2020). Alcohol use disorder and circulating cytokines: A systematic review and meta-analysis. Brain Behav Immun.

[64] Arab JP, Sehrawat TS, Simonetto DA, Verma VK, Feng D, Tang T (2020). An Open-Label, Dose-Escalation Study to Assess the Safety and Efficacy of IL-22 Agonist F-652 in Patients With Alcohol-associated Hepatitis. Hepatology.

[65] Hwang S, He Y, Xiang X, Seo W, Kim SJ, Ma J (2020). Interleukin-22 Ameliorates Neutrophil-Driven Nonalcoholic Steatohepatitis Through Multiple Targets. Hepatology.

[66] Xiang X, Feng D, Hwang S, Ren T, Wang X, Trojnar E (2020). Interleukin-22 ameliorates acute-on-chronic liver failure by reprogramming impaired regeneration pathways in mice. J Hepatol.

[67] Hwang S, Hicks A, Hoo CZ, Kwon YS, Cho YE, Moore J (2023). Novel treatment of acute and acute-on-chronic liver failure: Interleukin-22. Liver Int.

[68] He Y, Feng D, Hwang S, Mackowiak B, Wang X, Xiang X (2021). Interleukin-20 exacerbates acute hepatitis and bacterial infection by downregulating IκBζ target genes in hepatocytes. J Hepatol.

[69] Peeraphatdit TB, Simonetto DA, Shah VH (2018). Exploring new treatment paradigms for alcoholic hepatitis by extrapolating from NASH and cholestasis. J Hepatol.

[70] Aseem SO, Jalan-Sakrikar N, Chi C, Navarro-Corcuera A, De Assuncao TM, Hamdan FH (2021). Epigenomic Evaluation of Cholangiocyte Transforming Growth Factor-β Signaling Identifies a Selective Role for Histone 3 Lysine 9 Acetylation in Biliary Fibrosis. Gastroenterology.

[71] Cao S, Liu M, Sehrawat TS, Shah VH (2021). Regulation and functional roles of chemokines in liver diseases. Nat Rev Gastroenterol Hepatol.

[72] Dominguez M, Miquel R, Colmenero J, Moreno M, García-Pagán JC, Bosch J (2009). Hepatic expression of CXC chemokines predicts portal hypertension and survival in patients with alcoholic hepatitis. Gastroenterology.

[73] Affò S, Morales-Ibanez O, Rodrigo-Torres D, Altamirano J, Blaya D, Dapito DH (2014). CCL20 mediates lipopolysaccharide induced liver injury and is a potential driver of inflammation and fibrosis in alcoholic hepatitis. Gut.

[74] Liu M, Cao S, He L, Gao J, Arab JP, Cui H (2021). Super enhancer regulation of cytokine-induced chemokine production in alcoholic hepatitis. Nat Commun.

[75] Zannas AS, Wiechmann T, Gassen NC, Binder EB (2016). Gene-Stress-Epigenetic Regulation of FKBP5: Clinical and Translational Implications. Neuropsychopharmacology.

[76] Kusumanchi P, Liang T, Zhang T, Ross RA, Han S, Chandler K (2021). Stress-Responsive Gene FK506-Binding Protein 51 Mediates Alcohol-Induced Liver Injury Through the Hippo Pathway and Chemokine (C-X-C Motif) Ligand 1 Signaling. Hepatology.

[77] Wang X, He Y, Mackowiak B, Gao B (2021). MicroRNAs as regulators, biomarkers and therapeutic targets in liver diseases. Gut.

[78] Saha B, Momen-Heravi F, Kodys K, Szabo G (2016). MicroRNA Cargo of Extracellular Vesicles from Alcohol-exposed Monocytes Signals Naive Monocytes to Differentiate into M2 Macrophages. J Biol Chem.

[79] Eguchi A, Lazaro RG, Wang J, Kim J, Povero D, Willliams B (2017). Extracellular vesicles released by hepatocytes from gastric infusion model of alcoholic liver disease contain a MicroRNA barcode that can be detected in blood. Hepatology.

[80] Satishchandran A, Ambade A, Rao S, Hsueh YC, Iracheta-Vellve A, Tornai D (2018). MicroRNA 122, Regulated by GRLH2, Protects Livers of Mice and Patients From Ethanol-Induced Liver Disease. Gastroenterology.

[81] Heo MJ, Kim TH, You JS, Blaya D, Sancho-Bru P, Kim SG (2019). Alcohol dysregulates miR-148a in hepatocytes through FoxO1, facilitating pyroptosis via TXNIP overexpression. Gut.

[82] Luo J, Hou Y, Ma W, Xie M, Jin Y, Xu L (2021). A novel mechanism underlying alcohol dehydrogenase expression: hsa-miR-148a-3p promotes ADH4 expression via an AGO1-dependent manner in control and ethanol-exposed hepatic cells. Biochem Pharmacol.

[83] Li M, He Y, Zhou Z, Ramirez T, Gao Y, Gao Y (2017). MicroRNA-223 ameliorates alcoholic liver injury by inhibiting the IL-6-p47(phox)-oxidative stress pathway in neutrophils. Gut.

[84] Tang Y, Banan A, Forsyth CB, Fields JZ, Lau CK, Zhang LJ (2008). Effect of alcohol on miR-212 expression in intestinal epithelial cells and its potential role in alcoholic liver disease. Alcohol Clin Exp Res.

[85] Kumar V, Mansfield J, Fan R, MacLean A, Li J, Mohan M (2018). miR-130a and miR-212 Disrupt the Intestinal Epithelial Barrier through Modulation of PPARγ and Occludin Expression in Chronic Simian Immunodeficiency Virus-Infected Rhesus Macaques. J Immunol.

[86] Ramirez T, Li YM, Yin S, Xu MJ, Feng D, Zhou Z (2017). Aging aggravates alcoholic liver injury and fibrosis in mice by downregulating sirtuin 1 expression. J Hepatol.

[87] Ren R, He Y, Ding D, Cui A, Bao H, Ma J (2022). Aging exaggerates acute-on-chronic alcohol-induced liver injury in mice and humans by inhibiting neutrophilic sirtuin 1-C/EBPα-miRNA-223 axis. Hepatology.

[88] Huang M, Kong B, Zhang M, Rizzolo D, Armstrong LE, Schumacher JD (2020). Enhanced alcoholic liver disease in mice with intestine-specific farnesoid X receptor deficiency. Lab Invest.

[89] Donde H, Ghare S, Joshi-Barve S, Zhang J, Vadhanam MV, Gobejishvili L (2020). Tributyrin Inhibits Ethanol-Induced Epigenetic Repression of CPT-1A and Attenuates Hepatic Steatosis and Injury. Cell Mol Gastroenterol Hepatol.

[90] Agbu P, Carthew RW (2021). MicroRNA-mediated regulation of glucose and lipid metabolism. Nat Rev Mol Cell Biol.

[91] Dolganiuc A, Petrasek J, Kodys K, Catalano D, Mandrekar P, Velayudham A (2009). MicroRNA expression profile in Lieber-DeCarli diet-induced alcoholic and methionine choline deficient diet-induced nonalcoholic steatohepatitis models in mice. Alcohol Clin Exp Res.

[92] Wang W, Zhong GZ, Long KB, Liu Y, Liu YQ, Xu AL (2021). Silencing miR-181b-5p upregulates PIAS1 to repress oxidative stress and inflammatory response in rats with alcoholic fatty liver disease through inhibiting PRMT1. Int Immunopharmacol.

[93] Lohoff FW, Sorcher JL, Rosen AD, Mauro KL, Fanelli RR, Momenan R (2018). Methylomic profiling and replication implicates deregulation of PCSK9 in alcohol use disorder. Mol Psychiatry.

[94] Esfandiari F, Medici V, Wong DH, Jose S, Dolatshahi M, Quinlivan E (2010). Epigenetic regulation of hepatic endoplasmic reticulum stress pathways in the ethanol-fed cystathionine beta synthase-deficient mouse. Hepatology.

[95] Kim HG, Huang M, Xin Y, Zhang Y, Zhang X, Wang G (2019). The epigenetic regulator SIRT6 protects the liver from alcohol-induced tissue injury by reducing oxidative stress in mice. J Hepatol.

[96] Kim A, Wu X, Allende DS, Nagy LE (2021). Gene Deconvolution Reveals Aberrant Liver Regeneration and Immune Cell Infiltration in Alcohol-Associated Hepatitis. Hepatology.

[97] Weichselbaum L, Azouz A, Smolen KK, Das J, Splittgerber M, Lepida A (2020). Epigenetic basis for monocyte dysfunction in patients with severe alcoholic hepatitis. J Hepatol.

[98] Blaya D, Coll M, Rodrigo-Torres D, Vila-Casadesús M, Altamirano J, Llopis M (2016). Integrative microRNA profiling in alcoholic hepatitis reveals a role for microRNA-182 in liver injury and inflammation. Gut.

[99] Argemi J, Latasa MU, Atkinson SR, Blokhin IO, Massey V, Gue JP (2019). Defective HNF4alpha-dependent gene expression as a driver of hepatocellular failure in alcoholic hepatitis. Nat Commun.

[100] Albillos A, Martin-Mateos R, Van der Merwe S, Wiest R, Jalan R, Álvarez-Mon M (2022). Cirrhosis-associated immune dysfunction. Nat Rev Gastroenterol Hepatol.

[101] Kisseleva T, Brenner D (2021). Molecular and cellular mechanisms of liver fibrosis and its regression. Nat Rev Gastroenterol Hepatol.

[102] Chen X, Li WX, Chen Y, Li XF, Li HD, Huang HM (2018). Suppression of SUN2 by DNA methylation is associated with HSCs activation and hepatic fibrosis. Cell Death Dis.

[103] Mann J, Oakley F, Akiboye F, Elsharkawy A, Thorne AW, Mann DA (2007). Regulation of myofibroblast transdifferentiation by DNA methylation and MeCP2: implications for wound healing and fibrogenesis. Cell Death Differ.

[104] Page A, Paoli PP, Hill SJ, Howarth R, Wu R, Kweon SM (2015). Alcohol directly stimulates epigenetic modifications in hepatic stellate cells. J Hepatol.

[105] Clària J, Stauber RE, Coenraad MJ, Moreau R, Jalan R, Pavesi M (2016). Systemic inflammation in decompensated cirrhosis: Characterization and role in acute-on-chronic liver failure. Hepatology.

[106] Mezzano G, Juanola A, Cardenas A, Mezey E, Hamilton JP, Pose E (2022). Global burden of disease: acute-on-chronic liver failure, a systematic review and meta-analysis. Gut.

[107] Yang JD, Hainaut P, Gores GJ, Amadou A, Plymoth A, Roberts LR (2019). A global view of hepatocellular carcinoma: trends, risk, prevention and management. Nat Rev Gastroenterol Hepatol.

[108] Lambert MP, Paliwal A, Vaissière T, Chemin I, Zoulim F, Tommasino M (2011). Aberrant DNA methylation distinguishes hepatocellular carcinoma associated with HBV and HCV infection and alcohol intake. J Hepatol.

[109] Lu SC, Mato JM (2005). Role of methionine adenosyltransferase and S-adenosylmethionine in alcohol-associated liver cancer. Alcohol.

[110] Frau M, Feo F, Pascale RM (2013). Pleiotropic effects of methionine adenosyltransferases deregulation as determinants of liver cancer progression and prognosis. J Hepatol.

[111] Bayo J, Fiore EJ, Dominguez LM, Real A, Malvicini M, Rizzo M (2019). A comprehensive study of epigenetic alterations in hepatocellular carcinoma identifies potential therapeutic targets. J Hepatol.

